# Efficacy and safety of combined immune therapy for advanced cervical cancer: a systematic review and meta-analysis

**DOI:** 10.3389/fonc.2026.1772054

**Published:** 2026-05-25

**Authors:** Fan Shi, Yan Xuan, Yanling Zhu, Shangshu Wu, Xichen Sun, Dan Li

**Affiliations:** 1 Department of Gynecology, The First People’s Hospital of Lianyungang, Lianyungang, Jiangsu, China; 2 Department of Gynecology, The First Affiliated Hospital of Kangda College of Nanjing Medical University, Lianyungang, Jiangsu, China; 3 Department of Basic Medical Sciences, Kangda College of Nanjing Medical University, Lianyungang, Jiangsu, China; 4 Department of Gynecology, Xuzhou Cancer Hospital, Xuzhou, Jiangsu, China

**Keywords:** cervical cancer, efficacy, immune checkpoint inhibitor, immunotherapy, meta-analysis, safety

## Abstract

**Background:**

Combination therapy based on immune checkpoint inhibitors (ICIs) offer a viable treatment option for advanced cervical cancer. The effect of these treatments on patient survival is still unknown despite a large number of trials. The effectiveness and safety of ICI combination therapy for advanced cervical cancer are assessed in this study.

**Methods:**

The PRISMA guidelines were followed when conducting a meta-analysis. A thorough search for literature of four databases (PubMed, Web of Science, Embase, and Cochrane Library) yielded the results. ICI combinations and ICI-based dual combinations were the two types of regimens. Based on PD-L1 expression, baseline patient characteristics, and treatment plan, subgroup analysis was predetermined. The objective response rate (ORR), disease control rate (DCR), progression-free survival (PFS), overall survival (OS), 18-month PFS rate, treatment-related adverse events (TRAEs), and immune-related adverse events (irAEs) were among the outcomes of interest.

**Results:**

The analysis includes 18 trials with 4,441 participants. ICI combinations significantly improved OS (HR: 0.69; 95% CI: 0.60 - 0.78) and PFS (HR: 0.68; 95% CI: 0.61 - 0.75), and resulted in a higher ORR (RR: 1.15; 95% CI: 1.06 - 1.24) and DCR (RR: 1.04; 95% CI: 1.01 - 1.06). Dual ICI therapy did not show significant improvement in PFS at 12 months (RR: 1.26; 95% CI: 0.86-1.86), but demonstrated a notable improvement at 18 months (RR: 1.73; 95% CI: 1.04–2.87). The ORR and DCR were comparable between single and dual ICI regimens. Safety was generally well-managed, with a slight increase in adverse events for both combination regimens and dual ICIs. Subgroup analysis revealed that ICB combination therapy resulted in better PFS and OS for PD-L1+ patients, younger patients (< 65 years old), and those with squamous cell carcinoma histology.

**Conclusion:**

In patients with advanced cervical cancer, multi-agent regimens that combine chemotherapy, radiation, or targeted therapy with ICIs greatly enhance clinical results while reducing toxicity. Combined ICI therapy could offer a way to overcome resistance, even though it failed to vary significantly from monotherapy in regard to efficacy or safety—a feature that has to be validated by further randomized controlled trials.

**Systematic Review Registration:**

https://www.crd.york.ac.uk/PROSPERO, identifier CRD420251266881.

## 1 Introduction

Cervical cancer continues to be one of the biggest worldwide health issues affecting women, positioning it as a top priority in public health today. Treatment options for patients with advanced cervical carcinoma (ACC) include immunotherapy, in addition chemoradiotherapy, targeted therapy, and chemotherapy. However, clinical trial outcomes have varied, leaving the optimal therapeutic approach uncertain. Additionally, novel immunotherapies hold promising potential for patients with ACC, offering exciting opportunities for their integration into treatment strategies.

Immunotherapy development has primarily focused on PD-1/PD-L1 inhibitors. In 2020, the KEYNOTE-A18 study showed that pembrolizumab with radiation and chemotherapy was more effective than traditional chemoradiotherapy (1). In contrast, the CALLA trial in 2016 showed no significant improvement when combining durvalumab with concurrent chemotherapy (74). When compared to chemoradiotherapy alone, first-line therapies like sintilimab ± cetuximab in combination with chemotherapy and chemoradiotherapy for local ACC failed to reach the primary objectives of enhanced progression-free survival (PFS) or overall survival (OS) (2). Additionally, the CADON study (NCT02879724) demonstrated that cadonilimab combined with chemotherapy and targeted therapy did not significantly extend PFS or OS compared to placebo (3). The integration of findings from various clinical trials on immune combination therapies remains controversial, leaving the effectiveness of these treatments in ACC uncertain.

The safety and efficacy of PD-1/PD-L1 monotherapy or PD-1/PD-L1 inhibitors in combination with chemotherapy or other traditional therapies have been the focus of most meta-analyzes (4–6). However, given the plateauing efficacy of current standard therapies, investigating the therapeutic benefits of immune combination therapies in ACC is still worthwhile. In order to systematically assess the safety and effectiveness of novel immune combination treatment regimens, this study thoroughly searched and incorporated the most recent data from 2024 to 2025 (such as BEATcc, KEYNOTE-A18, and SKYSCRAPER-04). Clinical studies on immune combination therapies are exploring inhibitors targeting new checkpoints such as CTLA-4 and TIGIT, along with agents like bispecific cadonilimab (7, 8). This research provides a comprehensive summary of the positive and negative clinical benefits and safety profiles of immune combination therapies. The study also seeks to determine which patient subgroups could benefit most from these therapies. Such in-depth studies are essential for guiding clinicians in making informed decisions when treating patients, as well as optimizing treatment strategies for better outcomes.

## 2 Materials and methods

The protocol for the systematic review was given a number CRD420251266881 when it was submitted for prospective registration on PROSPERO, the global prospective systematic review registry. The PRISMA standards were strictly followed in the conduct of this study (9).

### 2.1 Search strategy

By August 30, 2025, two researchers (Fan Shi and Yan Xuan) had conducted complete electronic literature search across four databases: PubMed, Embase, Cochrane Library, and Web of Science.

MeSH phrases and free-text keywords were integrated in the search strategy. The category term “immune checkpoint inhibitors” and the specific agent names “pembrolizumab,” “nivolumab”, “atezolizumab”, “durvalumab”, “cemiplimab”, “camrelizumab”, “sintilimab”, “tislelizumab”, “toripalimab”, “avelumab”, “tremelimumab”, “ipilimumab”, “dostarlimab,” and “balstilimab” were included in the first search block. The Boolean operator OR was used to join these phrases. “Cervix,” “cervical cancer,” and “cervical neoplasms” were included in the cervical cancer component along with OR. AND was then used to merge the two blocks. There were no language or temporal restrictions. The whole search strategy is provided by the supplemental search terms (**Supplementary Table S1**).

The analysis excluded non-clinical research, systematic reviews, meta-analyses, and conference proceedings.

### 2.2 Inclusion and exclusion criteria

The study was chosen using the Population, Intervention, Comparison, Outcome (PICO) framework:

1. Population (P): ACC patients, including those with metastatic or recurring illness.
2. Intervention (I) and Comparison (C): As outlined in **Table 1**.
3. Outcome (O): Among the endpoints that were predetermined were: (1) OS, PFS, objective response rate (ORR), and disease control rate (DCR) were the major goals for randomized controlled trials (RCTs) of ICI combination therapy; treatment-related adverse events (TRAEs) and immune-related adverse events (irAEs) were the secondary endpoints; (2) For dual-ICI trials, primary endpoints were PFS, ORR, and DCR, with TRAEs as the secondary endpoint; (3) ORR was the primary endpoint and TRAEs were the secondary endpoint for ICI combination therapy single-arm trials.
4. Randomized controlled trials and single-arm studies were analyzed independently using meta-analysis in the analysis of studies on ICI-based combination therapies (such as ICI combined with concurrent chemoradiotherapy, ICI combined with chemotherapy and targeted therapy, ICI combined with radiotherapy, ICI combined with chemotherapy, and ICI combined with targeted agents). Since there aren’t enough pertinent RCTs in the field of dual ICI-based combination therapy, high-quality cohort studies were chosen using the Newcastle-Ottawa Scale and combined with RCTs for analysis. It should be noted that these combined findings should be regarded cautiously and are just meant to serve as an exploratory analysis in this field of study.

**Table 1:** Intervention and comparison.

NO	Intervention	Comparison
1	ICI Combined with Radiotherapy and Chemotherapy or ICI combined with chemotherapy and targeted agents.	Placebo Combined with Radiotherapy and Chemotherapy or Placebo combined with chemotherapy and targeted agents.
2	Dual ICI therapy	ICI monotherapy

### 2.3 Classification of included therapies

ICI-based combination treatment methods include ICI plus radiotherapy and chemotherapy, ICI plus chemotherapy and targeted therapy, ICI plus radiotherapy, ICI plus chemotherapy, and ICI plus targeted drugs.

Dual ICI therapy involves combining a monoclonal antibody that targets anti-PD-L1/PD-1 in conjunction with either an anti-TIGIT or an anti-CTLA-4 monoclonal antibody.

An anti-PD-L1/PD-1 monoclonal antibody is used in ICI monotherapy.

Chemotherapy alone, chemotherapy with targeted drugs, and chemotherapy plus radiation are examples of non-immune therapies (**Table 2**).

**Table 2:** Summary table of treatment plan.

Category	Treatment Regimen	Research Project
ICI-based combination treatment	ICI plus radiotherapy and chemotherapy	CALLA (2), KEYNOTE-A18 (1), NCT03298893 (23), PMC11447187 (24), NCT05084677 (25)
	ICI plus chemotherapy and targeted therapy	KEYNOTE-826 (18), BEATcc (17), COMPASSION-16 (3), COMPASSION-13 (21), NCT05247619 (28), NCT04973904 (29)
	ICI plus chemotherapy	NCT04864782 (22)
	ICI plus targeted drugs	NCT03816553 (26), NCT03827837 (27)
Dual ICI therapy	PD-L1/PD-1+TIGIT	SKYSCRAPER-04 (7),NCT04693234 (20)
	PD-L1+CTLA-4	NCT04590599 (19),NCT02488759 (8)

### 2.4 Data mining

The principal investigator’s specified inclusion and exclusion criteria were used to extract data from eligible studies, and a second reviewer confirmed the results. Extracted data included: (1) Study characteristics: clinical trial title, publication date, authors, registration number, country of origin, and study phase; (2) Baseline characteristics of the target population; (3) Clinical factors: treatment plans, PD-L1 expression level, and histologic subtype.

Efficacy evaluation for RCTs included outcomes such as OS, PFS, ORR, and DCR, with ORR as the primary outcome for single-arm studies. Tumor response assessment followed the RECIST 1.1 criteria (10). The CTCAE v3.0 criteria served as the basis for the safety evaluation (11). Both TRAEs and irAEs were analyzed, with outcomes summarized narratively and referenced in **Supplementary Tables** (**Supplementary Tables S2**, **S3**).

### 2.5 Quality assessment

Cochrane’s Risk of Bias assessment for randomized trials (RoB 2) was used to evaluate the possible risk of bias in the chosen RCTs (12, 13). The Newcastle-Ottawa Scale (NOS) for cohort and single-arm studies (14) and the ROBINS-I tool for single-arm cohort studies were used to evaluate bias risk (15).

### 2.6 Statistical analysis and subgroup stratification strategy

Survival and safety data were extracted directly from the original publications, with survival data from several studies obtained from published Kaplan–Meier curves. Heterogeneity was evaluated using I2 and χ2 test statistics: fixed-effects modeling was applied when I2 ≤ 50% and χ2 > 0.1, while random-effects models were used in other cases. A meaningful p-value was defined as one that was below 0.05 (16). Funnel plots were used to visually assess potential publication bias, and Egger’s regression test was used to formally evaluate it.

Stata 18.0 and Review Manager 5.4 (V.5.4.7) were utilized for statistical analysis. To address variations in populations and reduce clinical and methodological heterogeneity from merging data across studies in the meta-analysis, subgroup analyzes were performed based on PD-L1 expression levels, patient baseline characteristics prior to treatment, and treatment regimens, thereby strengthening the sequence of evidence.

## 3 Results

### 3.1 Searching results

**Figure 1:**
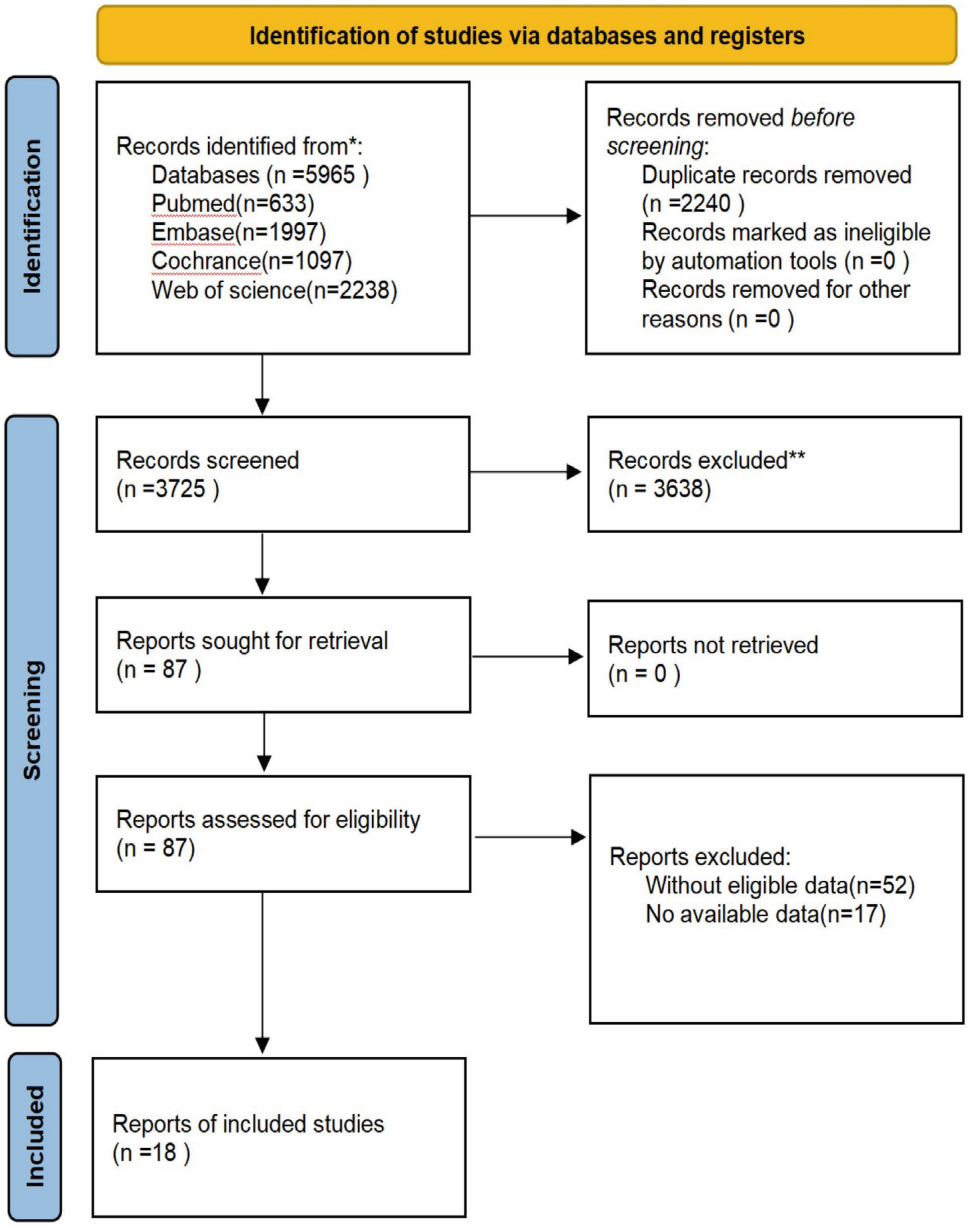
PRISMA flowchart of the screening and inclusion process.

The PRISMA flowchart describes the research selection process. Out of the 3,684 candidates discovered by a systematic search of electronic medical databases, 18 papers were chosen for both the systematic review and meta-analysis following a rigorous screening and selection process using EndNote (**Figure 1**).

### 3.2 Study attributes

The analysis included 18 publications, encompassing 4,441 patients. The corpus consisted of: five RCTs, including 3,302 patients (1–3, 17, 18); three Phase II trials, including 554 patients (7, 19, 20); two multi-cohort studies, including 261 patients (8, 21); and eight single-arm trials, including 349 patients (22–29). PD-L1 inhibitors, PD-1 inhibitors, cadonilimab (a bispecific antibody), CTLA-4 inhibitors, and TIGIT inhibitors were among the immune checkpoint inhibitors that were assessed.

### 3.3 Efficacy

#### 3.3.1 ICIs combined therapy versus non-immune therapy for OS/PFS in ITT patients

**Figure 2:**
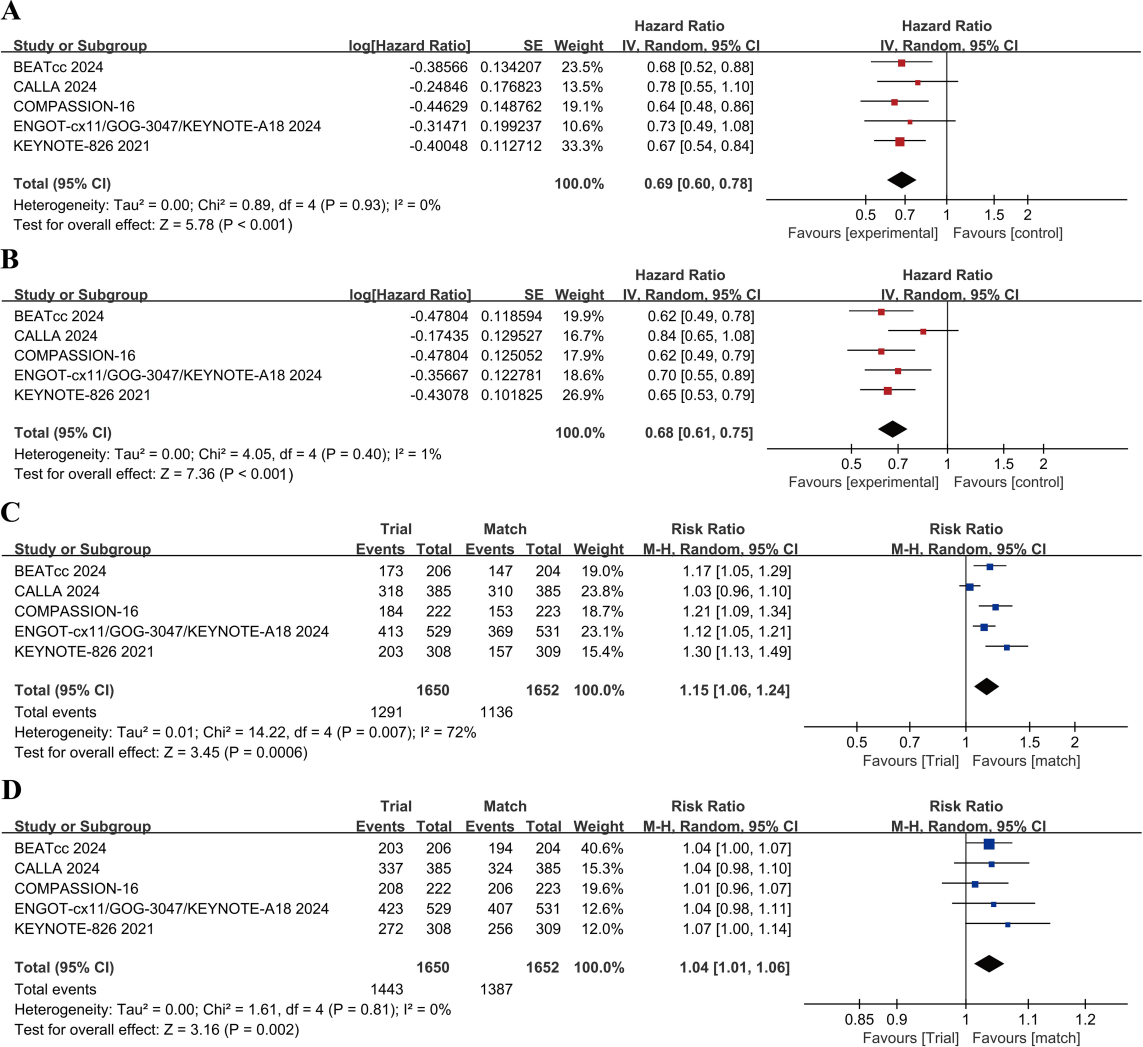
Forest plot of efficacy outcomes from studies evaluating immune checkpoint inhibitor-based combination therapy. **(A)** OS in RCTs; **(B)** PFS in RCTs; **(C)** ORR in RCTs; **(D)** DCR in RCTs; **(E)** ORR in Single-Arm Studies.

Five RCTs with 3,302 OS individuals were included in the study. These studies showed no heterogeneity (I^2^ = 0%). With a pooled HR of 0.69 (95% CI: 0.60–0.78; **Figure 2A**), ICI-based combination treatment considerably extended OS in comparison to non-immunotherapies. With a maximal 2-year OS rate of up to 87%, the combination treatment group demonstrated a 31% decrease in the risk of mortality.

For PFS, no heterogeneity (I^2^ = 1%) was found in the subgroup analysis, which showed superiority of all ICI-based combination regimens compared to non-immune therapy. A 32% reduction in the risk of either death or disease progression was shown by the SA pooled HR of 0.68 (95% CI: 0.61–0.75; **Figure 2B**). The highest reported 2-year PFS for the combination therapy was 69.1%.

#### 3.3.2 ICI-based combination therapy versus non-immune therapy in the ITT population: ORR and DCR

The pooled analysis of ORR revealed significant heterogeneity (I^2^ = 72%). **Figure 2C** illustrates that the ORR in the ICI-based combination treatment group was considerably greater than in the non-immune group (RR 1.15, 95% CI: 1.06–1.24).

For DCR, minimal heterogeneity was observed (I^2^ = 0%). Furthermore, with a pooled RR of 1.04, the ICI-based combination therapy group showed noticeably better DCR than other regimens (95% CI: 1.01–1.06; **Figure 2D**).

#### 3.3.3 Dual ICI therapy versus ICI monotherapy in the ITT population: PFS

**Figure 3:**
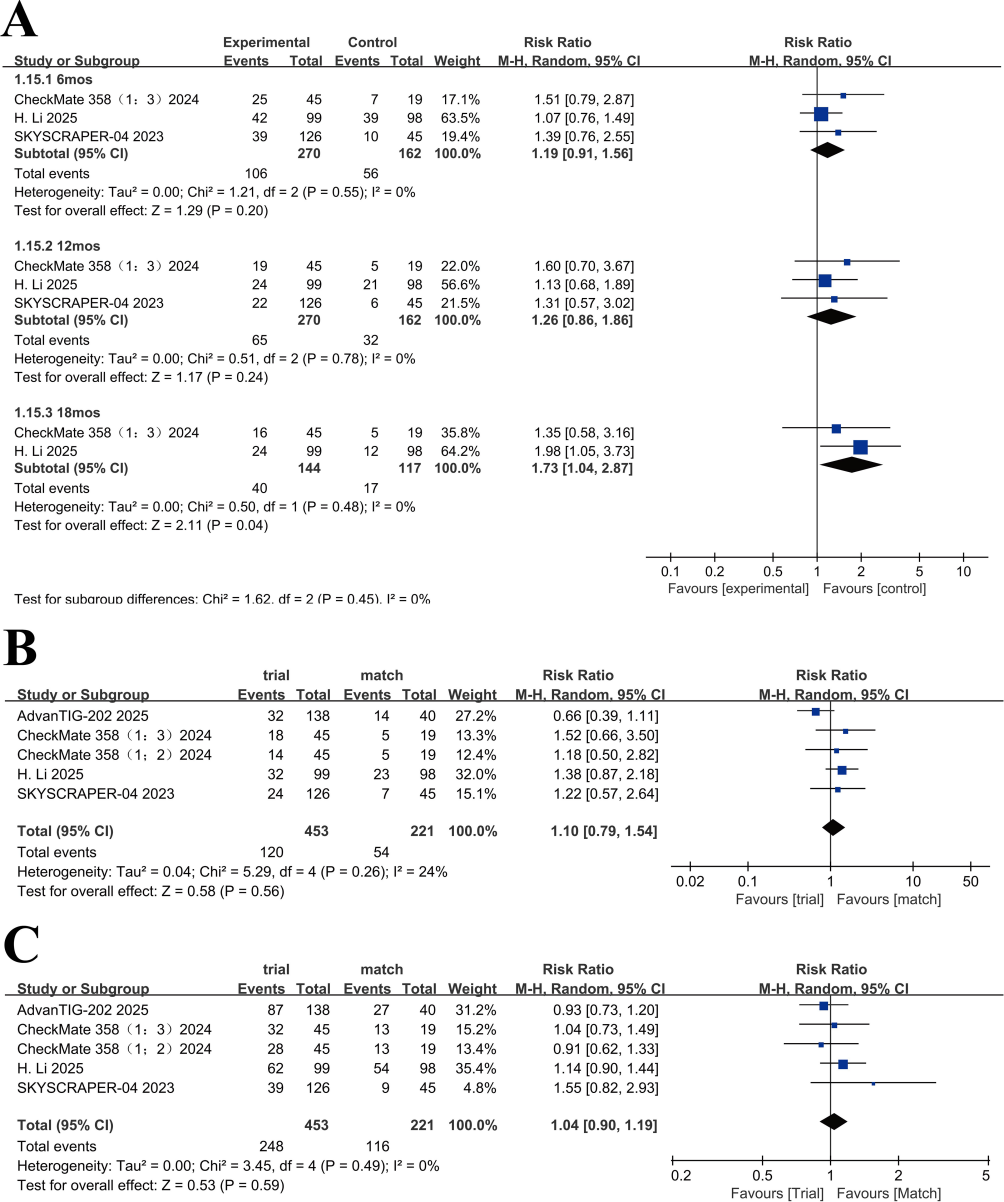
Forest plot of efficacy outcomes from randomized controlled trials evaluating dual immune checkpoint inhibitor therapy. **(A)** PFS; **(B)** ORR; **(C)** DCR.

PFS analysis combined three Phase II studies and one multi-cohort comparative study, totaling 775 patients. The pooled analysis of PFS showed no difference between combined dual ICIs and monotherapy at six months (RR: 1.19, 95% CI: 0.91–1.56, **Figure 3A**), or at 12 months (RR: 1.26, 95% CI: 0.86–1.86, **Figure 3A**). However, a notable difference was observed at the 18-month mark (RR: 1.73, 95% CI: 1.04–2.87, **Figure 3A**).

#### 3.3.4 Dual ICI therapy versus ICI monotherapy in the ITT population: ORR and DCR

Meta-analysis showed that the objective ORR for dual ICI was not significantly superior compared to monotherapy (RR: 1.10, 95% CI: 0.79–1.54), as shown in **Figure 3B**. Similarly, there was no significant distinction in DCR between dual and single ICI (RR: 1.04, 95% CI: 0.90–1.19), as shown in **Figure 3C**. Most of the trials that were included had quite small sample sizes, which might help to explain why dual ICI treatment did not significantly improve ORR and DCR.

#### 3.3.5 Single-arm studies of ICI-based combination therapy in the ITT population for ORR

The analysis comprised 349 patients from one multi-cohort study and eight single-arm trials. The pooled RD was 0.78 (95% CI: 0.64–0.88; **Supplementary Figure S4**) in the meta-analysis on ORR, indicating that, as expected, ICI combination therapy was more effective in improving ORR.

### 3.4 Safety

In order to analyze the safety profile of ICI-based combination therapy, the frequency of all-grade TRAEs and irAEs was determined using two crucial parameters: the percentage of Grade 3–4 or higher serious adverse events and the total occurrence of adverse reactions.

#### 3.4.1 Effectiveness of ICI-based combination therapy against non-immune therapy in ITT patients regarding TRAEs and irAEs

**Figure 4:**
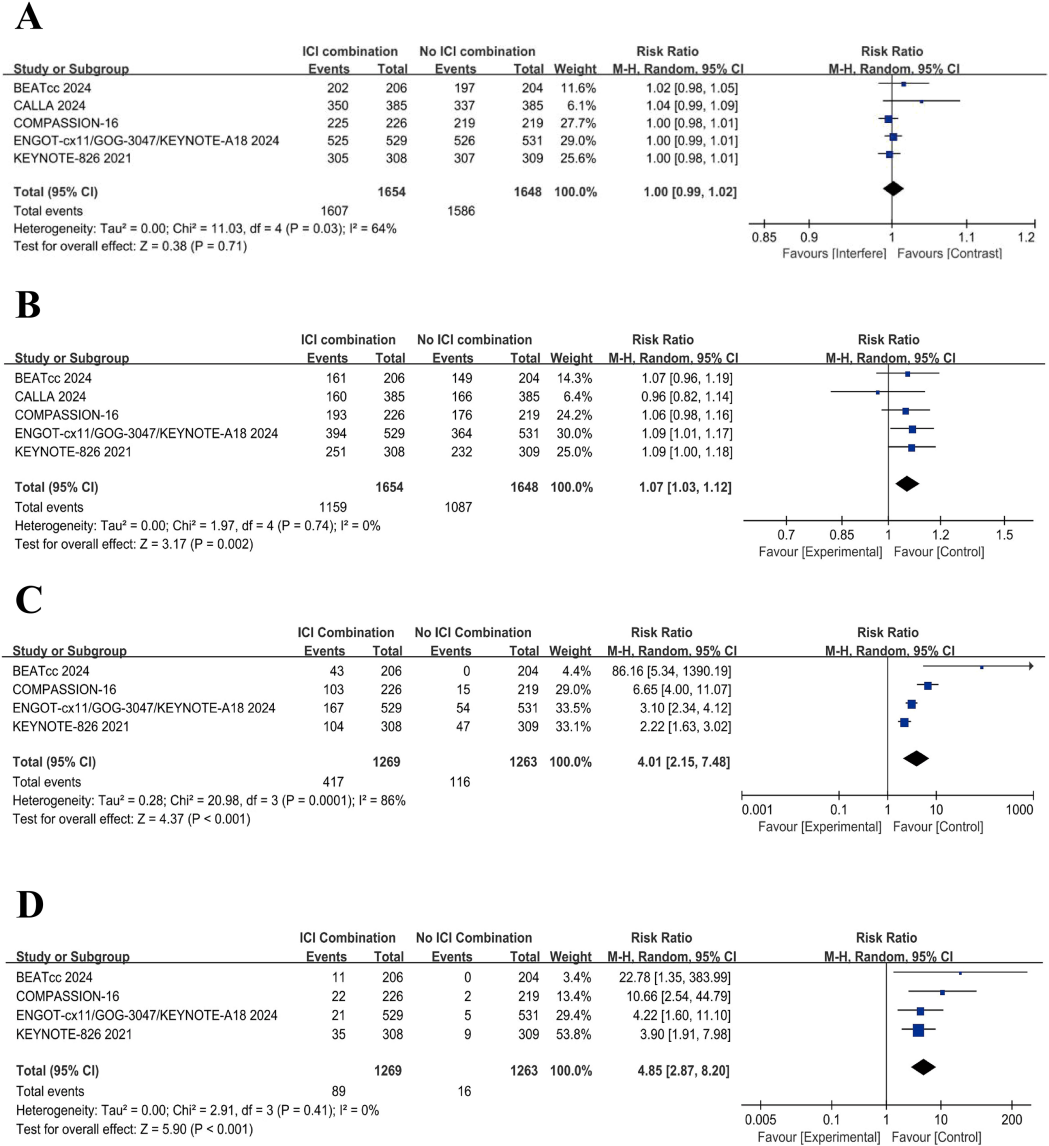
Forest plot of safety outcomes from studies evaluating immune checkpoint inhibitor-based combination therapy. **(A)** Adverse Events of Any Grade in RCTs; **(B)** ≥G3 AEs in RCTs; **(C)** Immune-related adverse events of Any Grade in RCTs; **(D)** Immune-related adverse events of Grade 3–5 in RCTs.

The analysis of five RCTs with 3,302 participants revealed no statistically significant distinction in TRAE occurrence rates when comparing ICI-ICI combination therapy to non-immune therapy. However, compared to those without ICI, the combination therapy groups had a somewhat higher incidence of Grade 3–5 TRAEs, with an RR of 1.07 (95% CI: 1.03–1.12; **Figure 4B**). Clinical study results demonstrated that risk estimates for any-grade and Grade 3–5 irAEs were significantly greater in combination treatment groups compared to those without ICI (RR = 4.01, 95% CI: 2.15–7.48, **Figure 4C**; RR = 4.85, 95% CI: 2.87–8.20, **Figure 4D**).

#### 3.4.2 Dual ICI therapy versus ICI monotherapy in the ITT population: TRAEs

**Figure 5:**
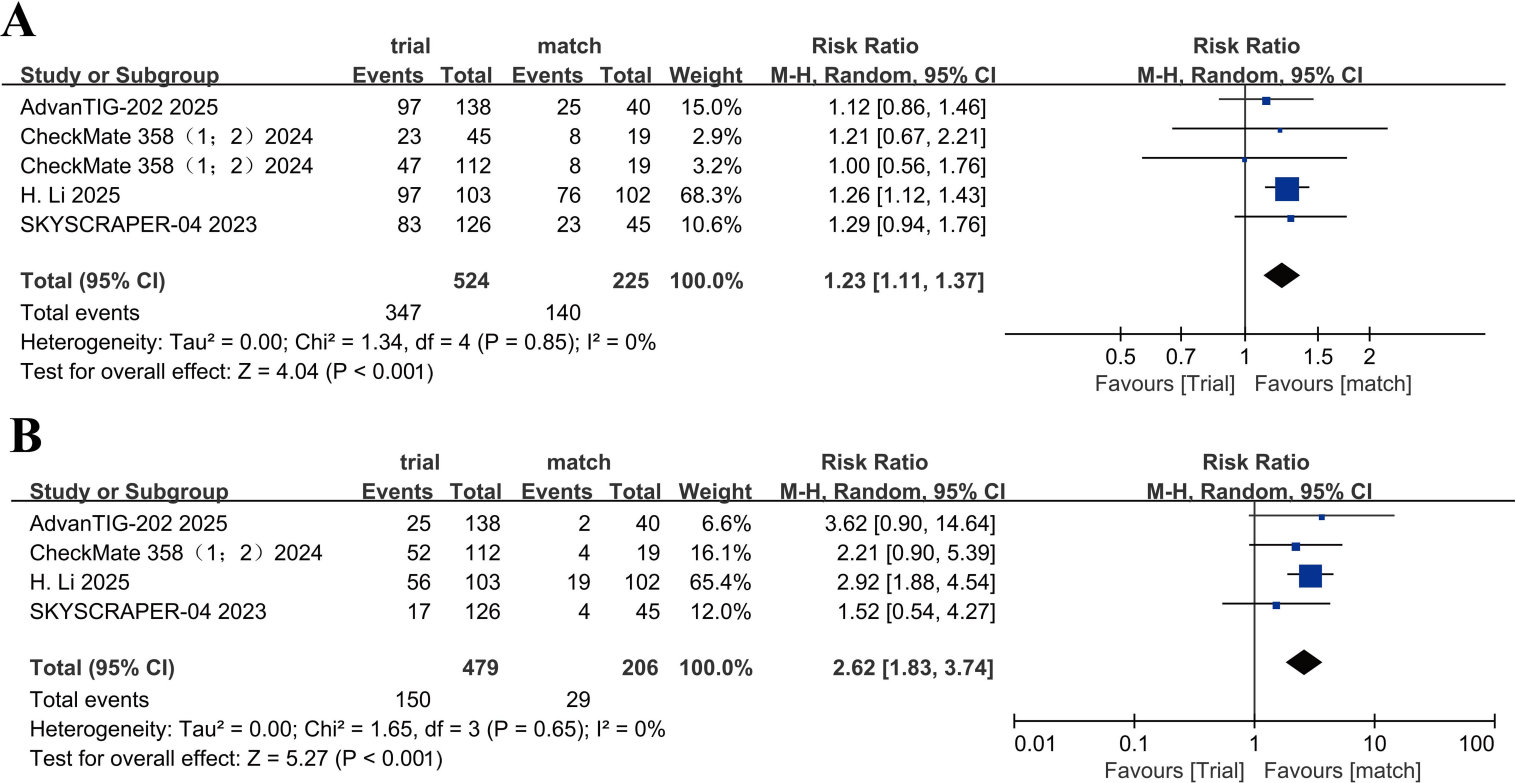
Forest plot of safety outcomes from randomized controlled trials evaluating dual immune checkpoint inhibitor therapy. **(A)** Adverse events of any grade; **(B)** ≥ G3 AEs.

The analysis included three Phase II studies, with 775 patients, one multicohort comparative study, and one meta-analysis of TRAEs. A significant risk of TRAEs of any grade was found in the dual ICI group (RR = 1.23, 95% CI: 1.11–1.37, **Figure 5A**), as well as an increased risk of Grade 3–5 TRAEs (RR = 2.62, 95% CI: 1.83–3.74, **Figure 5B**) when compared to the single-agent ICI group.

#### 3.4.3 Single-arm studies of ICI-based combination therapy: TRAEs in the ITT population

One multicohort study and six single-arm trials were examined. High frequencies of all-grade TRAEs were found in meta-analyses (0.96, 95% CI: 0.93-0.98, **Supplementary Figure S5**).

### 3.5 Subgroup analysis

#### 3.5.1 Level of PD-L1 expression

**Figure 6:**
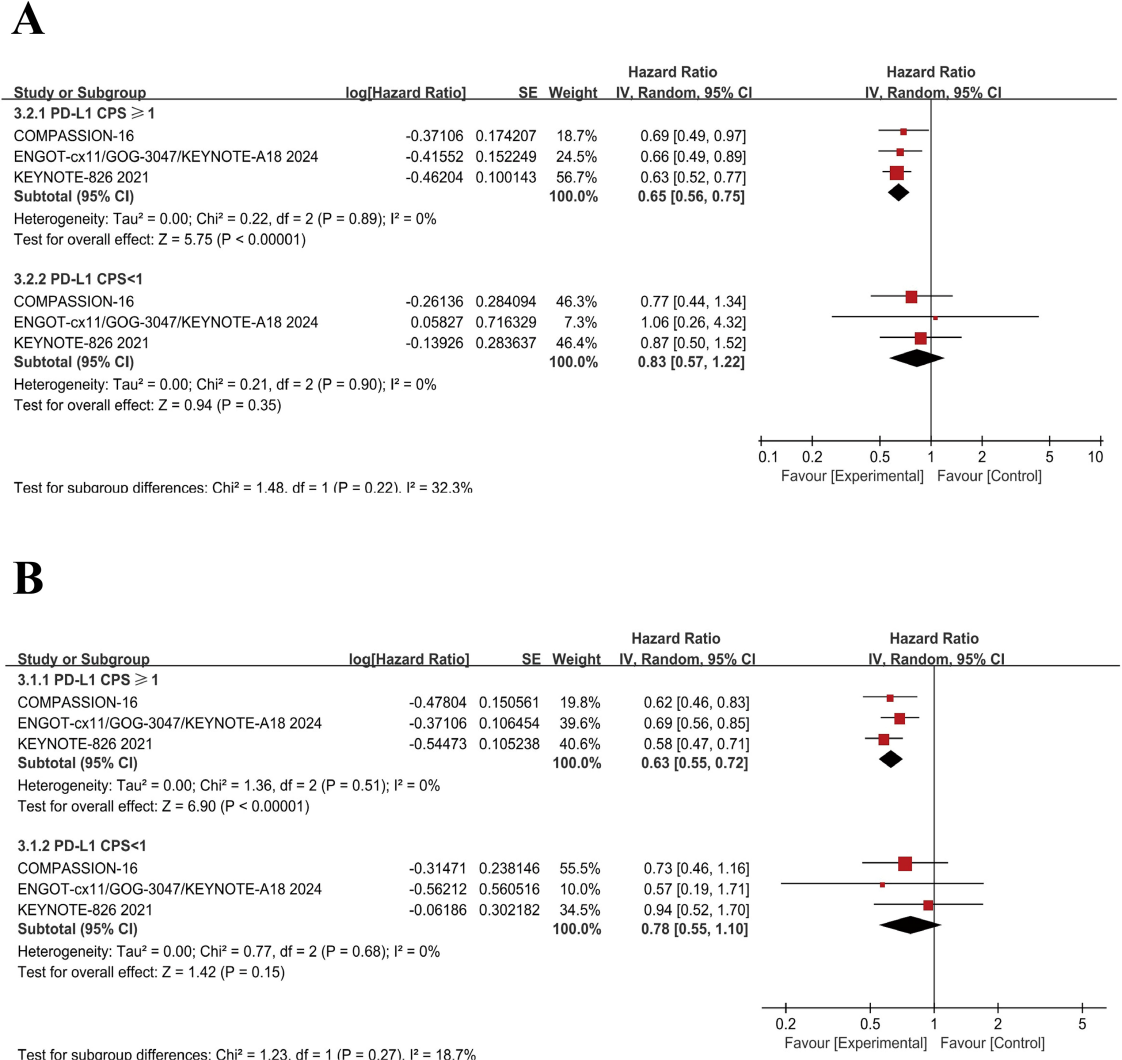
Forest plots for subgroup analysis by PD-L1 expression status on treatment efficacy in randomized controlled trials. **(A)** OS in RCTs; **(B)** PFS in RCTs.

PD-L1’s potential as a diagnostic tool for ICI-CT-based combination therapy was investigated by additional subgroup research according to PD-L1 expression levels. To assess therapy effectiveness, patients were categorized based on PD-L1 combined positive score (CPS) thresholds (≥1 vs. <1).Combination therapy performed better than ICI treatment in the PD-L1 CPS≥1 subgroup in terms of OS (HR = 0.65, 95% CI: 0.56–0.75, **Figure 6A**) and PFS (HR = 0.63, 95% CI: 0.55–0.72, **Figure 6B**). However, OS (HR = 0.83, 95% CI: 0.57–1.22, **Figure 6A**) and PFS (HR = 0.78, 95% CI: 0.55–1.10, **Figure 6B**) did not differ statistically significantly in the PD-L1 CPS<1 subgroup (30, 31).

#### 3.5.2 Patient characteristics

To explore why some patients benefited more from this treatment, its effect was assessed in four subgroups: age, presence or absence of metastasis, histology, and ECOG status.

**Figure 7:**
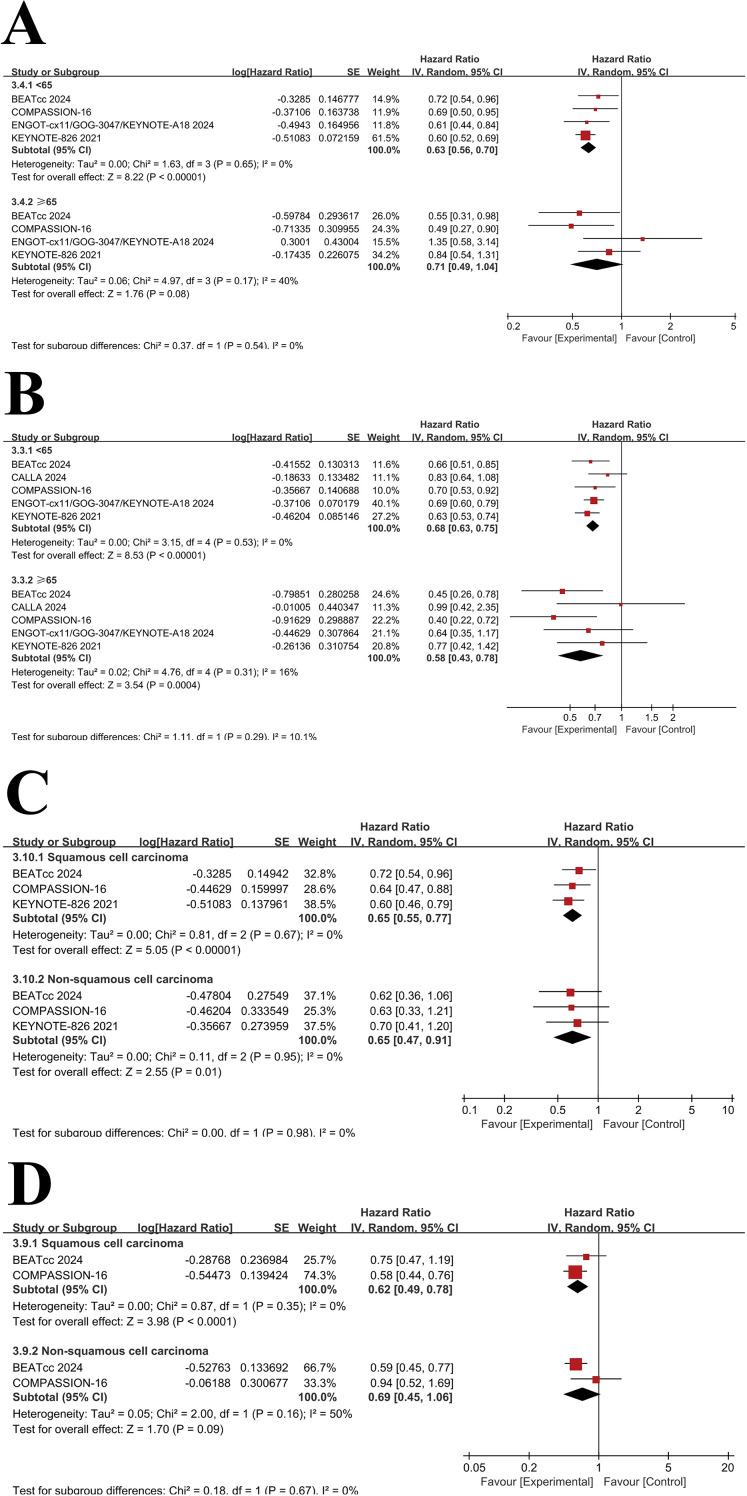
Forest plots of subgroup analysis categorized by clinical characteristics: patient characteristics. **(A)** Age OS in RCTs; **(B)** Age PFS in RCTs; **(C)** Histology OS in RCTs; **(D)** Histology PFS in RCTs.

ICI-based combination therapy increased OS (HR = 0.63, 95% CI: 0.56–0.70, **Figure 7A**) and PFS (HR = 0.68, 95% CI: 0.63–0.75, **Figure 7B**) in patients under 65. Conversely, OS did not demonstrate a statistically significant improvement (HR = 0.71, 95% CI: 0.49–1.04, **Figure 7A**), although PFS significantly improved in patients 65 years of age or older. A substantial difference in the effects of OS treatment between these two age categories was confirmed by an interaction test.

In subgroup analyzes based on ECOG status and metastasis, PFS and OS improved in the ICI-based combination therapy group, regardless of ECOG status (0 vs 1) or metastatic disease presence (yes vs no). **Supplementary Figures S1** and **S2** display corresponding forest plots.

In histology-based subgroup analyzes, favorable effects on OS were observed in both the squamous cell carcinoma (HR = 0.65, 95% CI: 0.55–0.77, **Figure 7C**) and non-squamous cell carcinoma (HR = 0.65, 95% CI: 0.47–0.91, **Figure 7C**) groups. PFS significantly improved only in patients with squamous cell carcinoma (HR = 0.62, 95% CI: 0.49–0.78, **Figure 7D**); non-squamous cell carcinoma patients had no significant gains (HR = 0.69, 95% CI: 0.45–1.06, **Figure 7D**).

#### 3.5.3 Treatment regimen

The combination of ICI, chemotherapy, and bevacizumab significantly improved both OS (HR = 0.67, 95% CI: 0.57–0.77) and PFS (HR = 0.63, 95% CI: 0.56–0.72) when compared to controls, according to an analysis of outcomes stratified by particular treatment regimens. Similarly, the combination of ICI with chemotherapy and radiotherapy also showed better outcomes than the control group in terms of OS (HR = 0.76, 95% CI: 0.64–0.91) and PFS (HR = 0.76, 95% CI: 0.58–0.98). **Supplementary Figure S3** displays pertinent forest plots.

**Figure 8:**
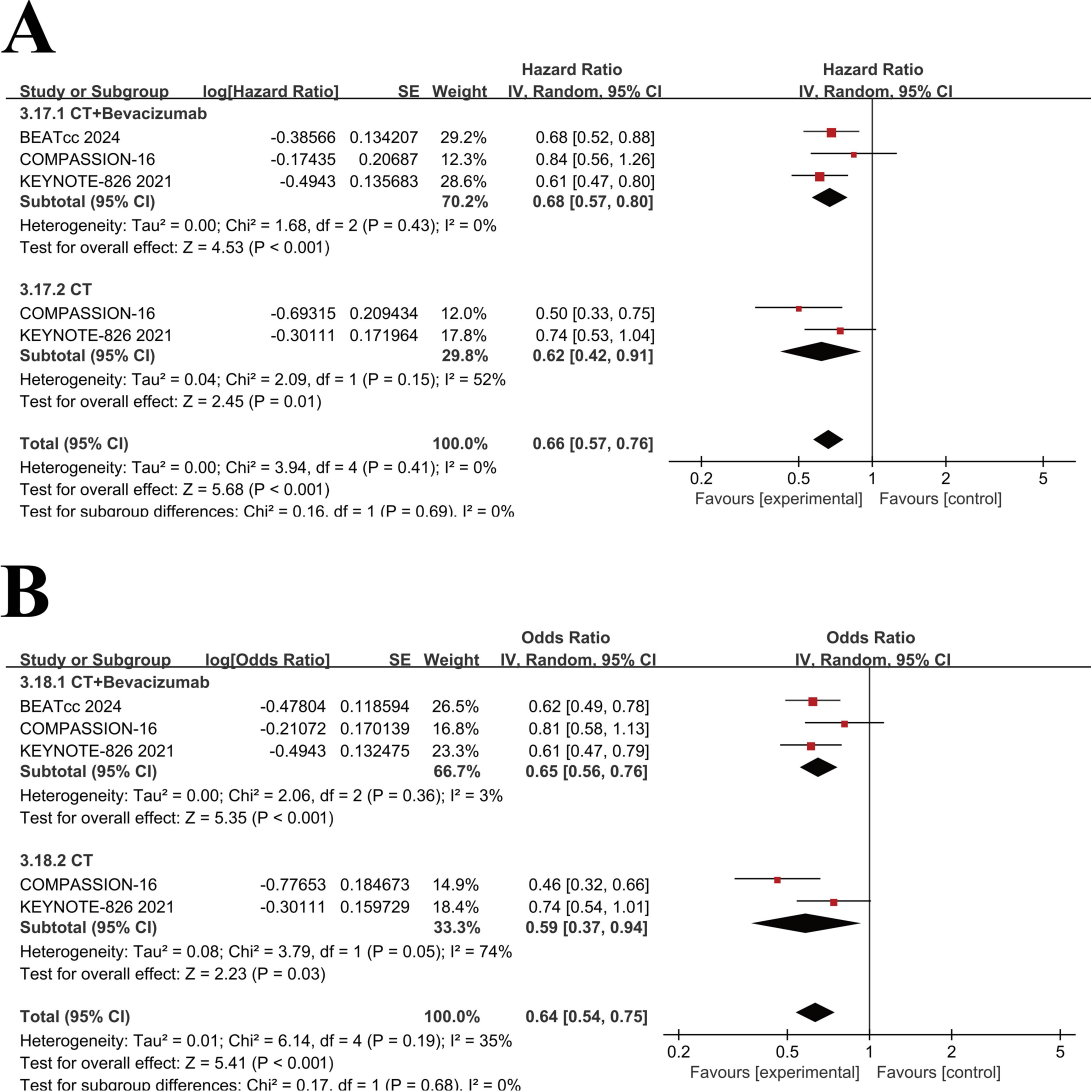
Forest plots of subgroup analysis categorized by clinical characteristics: treatment regimen. **(A)** OS in RCTs; **(B)** PFS in RCTs.

ICI combinations with chemotherapy, including bevacizumab, showed a statistically significant distinction in efficacy compared to those without. Both OS (HR = 0.68, 95% CI: 0.57–0.80; **Figure 8A**) and PFS (HR = 0.65, 95% CI: 0.56–0.76; **Figure 8B**) were significantly improved with ICI + chemotherapy regimens such as ColoxFAC/Tol and icotecel/chiroxlrBlz. Similarly, both OS (HR = 0.62, 95% CI: 0.42–0.91; **Figure 8A**) and PFS (HR = 0.59, 95% CI: 0.37–0.94; **Figure 8B**) were significantly enhanced by ICI + chemotherapy regimens not containing bevacizumab.

### 3.6 Heterogeneity and sensitivity analysis

**Figure 9:**
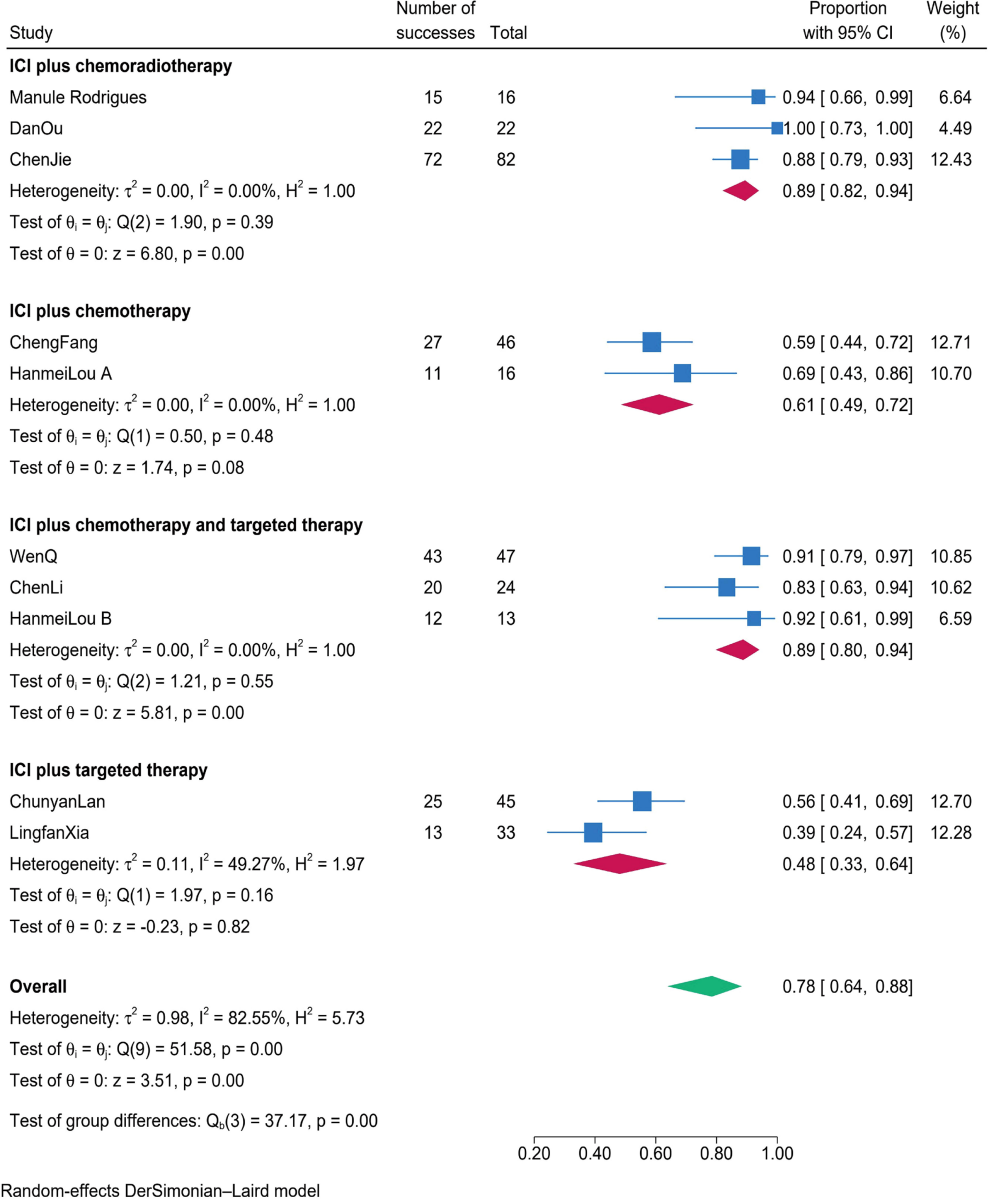
Forest plots of subgroup analysis categorized by clinical characteristics: treatment regimen. ORR in single-arm studies.

The I2 statistic was used to evaluate heterogeneity. The immune combination therapy randomized controlled trial group showed significant heterogeneity, with I² values of 72% for objective response rate (ORR), 64% for treatment-related adverse events (TRAEs), and 86% for immune-related adverse events (irAEs). In the single-arm analysis, heterogeneity was likewise considerable for ORR. Pre-specified subgroup analyses were performed for insight into potential causes of heterogeneity, and treatment regimen was found to be a significant contributing factor. The ICI plus chemoradiotherapy subgroup in the single-arm trials subgroup had significantly less ORR heterogeneity (I² = 0%), but the ICI plus targeted drugs subgroup still had a moderate amount of heterogeneity (I² = 49.27%) (**Figure 9**). Variations in patient baseline characteristics, methodological variability between studies, and differences in cancer kind and stage may be other causes of heterogeneity. Notably, sensitivity analyses for ORR, TRAEs, and irAEs showed that the pooled effect estimates did not change direction after sequential omission of individual studies, and the results remained robust after excluding studies at high risk of bias, suggesting that the study’s main conclusions are trustworthy despite the significant heterogeneity (**Supplementary Figure S6**).

### 3.7 Risk of bias assessment

The ROB-2 (**Supplementary Figure S9**), ROBINS-I (**Supplementary Table S5**), and Newcastle-Ottawa Scale (NOS) instruments (**Supplementary Table S4**) were used to evaluate the risk of bias. Five of the six included randomized controlled studies used a double-blind design, while one was open-label. The open-label trial has a high risk of performance bias and detection bias, but low risks of selection bias, attrition bias, and reporting bias, according to the RoB 2 risk of bias assessment tool. The remaining five trials were deemed to have a minimal risk of bias. Four studies were classified as low risk using the NOS (7, 8, 20, 21). Four single-arm trials were categorized as low risk (23, 25–27) and four as moderate risk (22, 24, 28, 29) using ROBINS-I. Egger’s test revealed possible publication bias for all-cause grade 3–5 adverse events (P = 0.035) and objective response rate (P = 0.034). The robustness of the results was confirmed by the trim-and-fill correction, which produced pooled estimates that were consistent in direction and significance with the initial analysis. Other results showed no evidence of publication bias (**Supplementary Figures S7**, **S8**).

## 4 Discussion

### 4.1 Summary of findings of the meta-analysis and systematic review

Clinicians continue to disagree about the safety and effectiveness of mixing ICIs. To assess the efficacy of RCTs, Phase II controlled trials, multi-cohort studies, and single-arm trials, a thorough review and meta-analysis were carried out. These 18 trials were used to establish the safety and effectiveness characteristics of ICIs.

In the context of ICI-based combination treatment for advanced-stage cervical cancer, the results indicate that although combination immunotherapy regimens offer improved therapeutic efficiency, they may also pose additional safety concerns, with only marginal improvements in efficacy outcomes. Dual ICI therapy is not significantly superior to single-agent ICI in short-term efficacy assessments, but it better maintains tumor control and the anti-tumor effect over time.

The meta-analysis of RCTs showed that ICI-based combination therapy—defined as ICI plus chemotherapy with or without bevacizumab—produced significant survival benefits, with pooled hazard ratios of 0.70 (95% CI: 0.61–0.81) for OS and 0.70 (95% CI: 0.62–0.80) for PFS. Additionally, significant improvements were observed in the ORR (RR 1.15, 95% CI: 1.06–1.24) and DCR (RR 1.04, 95% CI: 1.01–1.06). These results show that patients with ACC may benefit significantly from combination therapy regimens. From a mechanistic perspective, the administration of multiple agents may induce immunogenic cell death (ICD) in cervical cancer cells, releasing DAMPs that activate both adaptive and innate immunity.

Tumor antigens presented by mature DCs lead to increased infiltration and activation of CTLs, which initiate a strong antitumor immune response. This response can synergistically combine with ICIs to enhance tumor cell killing through a coordinated “ICD-DAMPs immune activation-ICI potentiation” circuit. Furthermore, these combined approaches can transform the tumor microenvironment (TME) into an immunogenic one, making other combination regimens more effective.

Pushing “cold” tumors to adopt ICI-responsive phenotypes offers significant expansion of patient populations that can benefit from immunotherapy (32–34), as validated by the KEYNOTE-826 trial, which supports the superiority of combined immunotherapy regimens over monotherapy for treating metastatic cervical cancer (35). The pooled ORR (RD = 0.72, 95% CI: 0.61–0.84) was observed in the meta-analysis of single-arm studies.

The clinical activity of these regimens is significant, with promising combinations, such as ICI in association with TKIs, showing particular potential. Representative multitarget TKIs—apatinib, anlotinib, and famitinib—can suppress tumor angiogenesis by blocking receptors such as VEGFR and PDGFR. They also notably transform the immunosuppressive TME, providing a possible therapeutic approach to enhance the prognosis of patients with advanced cervical tumors that has spread (36).

Anti-PD-L1/PD-1 and anti-TIGIT/anti-CTLA-4 monoclonal antibody combinations are the main components of dual ICI treatments. In a range of solid tumors, dual checkpoint blockade using nivolumab (anti-PD-1) + ipilimumab (anti-CTLA-4) has demonstrated superior clinical results compared to anti-PD-1 monotherapy. Tumor-reactive T cells functionally recover when the PD-1 pathway is blocked.

CTLA-4 inhibition specifically reduces Tregs in the TME while enhancing the activation of CD4+ and CD8+ effector cells. Compared to single-agent immune checkpoint inhibitors, dual ICI treatment may improve the synergy between antitumor immunity and tumor rejection, perhaps resulting in higher efficacy. Due to their mutually exclusive expression, dual ICI therapy may mitigate the acquired resistance often observed in ICI monotherapy. The synergy between dual ICIs can effectively prevent *de novo* resistance to immunotherapy in the CC treatment group (37, 38).

Dual blockade of TIGIT and PD-1 is currently a developing strategy that improves the effector capabilities of both CD8+ T cells and NK cells by simultaneously inhibiting TIGIT and PD-L1. Recent studies have observed that CD96, a member of the TIGIT axis predominantly expressed on T and NK cells, functions synergistically with the PD-1 pathway to impair the activity of CD8+ tumor-infiltrating lymphocytes (TILs). This has been noted in cervical carcinoma cells. Therefore, combining anti-PD-1 and anti-TIGIT treatments, which interfere with both the CD96 and PD-1 pathways. Therefore, the antitumor activity of CD8+ TILs may be increased (38, 39). Although multi-faceted immunological evidence supports the promising results of dual ICIs, meta-analysis of integrated multi-mechanism data revealed significant heterogeneity between studies and did not demonstrate increased benefits from dual ICI therapy.

The data showed no significant enhancement of ORR or DCR over ICI monotherapy, indicating a discordance between expected and real-world efficacy outcomes. We hypothesize that the lack of randomized controlled trials (RCTs) in the field of dual immune checkpoint inhibitor (dual ICI) therapy may be due to the small sample size overall and the small number of dual ICI studies included, making it challenging to identify possible slight variations in treatment effect. Furthermore, the NCT04590599 study’s limitations revealed that the observed objective response rate (ORR) in the placebo plus sintilimab arm was higher than expected. This could be one of the reasons why the combination of cetuximab and IBI310 did not show a significant improvement in ORR with the current sample size (19). Therefore, it is still unclear if dual ICI therapy may have the hypothetical synergistic benefit, and more research using large-scale randomized controlled trials is necessary to validate this. In terms of safety, compared to ICI monotherapy, dual ICI therapy was linked to a greater rate of TRAEs, consistent with a previously conducted RCT comparing the safety and efficacy of IBI310 plus sintilimab (19). However, no significant reduction in disease progression (i.e., the risk of tumor enlargement) was observed.

It was unclear whether OS differed within the first year (RR = 1.26, 95% CI: 0.86–1.86), but extending the follow-up to 18 months showed statistically significant benefits in PFS (RR = 1.73, 95% CI: 1.04–2.87). The delayed and enduring effects align with results from CheckMate 358, suggesting that dual ICI therapy may overcome resistance encountered with monotherapy and persist long-term to exhibit sustained anti-tumor effects (8). Larger randomized trials are necessary to confirm the encouraging findings found, even with the small sample size.

Although the control group in RCTs had a greater incidence of TRAEs, safety analysis did not find a meaningful difference in TRAEs between patients receiving ICI combination therapy and the control group. Patients in dual-ICI clinical trials experienced a further increase in adverse events. Safety concerns should be heightened during clinical management of such patients, with strategies to improve the management of adverse events. Additionally, close observation and intervention for irAEs are crucial, including discontinuation of treatment and co-administration of immunosuppressants (e.g., infliximab, tocilizumab). Tailored management is essential for addressing individual irAEs associated with combination immunotherapy.

Age, histology, and PD-L1 expression all had a substantial impact on treatment outcomes, according to subgroup analyses Patients with PD-L1 CPS ≥ 1, those with squamous cell carcinoma histology showed the biggest advantages from ICI combination therapy. For elderly individuals over 65, immunological combination treatment is not as effective as it may be. We speculate that immunosenescence decreases CD8+ T cell infiltration and functioning in addition to impairing the host’s capacity to manage malignancies. Concurrently, the ability of conventional type 1 dendritic cells (cDC1) to stimulate CD8+ T cells is reduced due to aging-related malfunction. When taken as a whole, these variables may be responsible for the decreased effect shown in older cancer patients using these immunotherapeutic techniques (40). Moreover, the antitumor effectiveness of anti-PD-1 and anti-CTLA-4 immunotherapy is strongly correlated with the numbers of systemic naive CD8+ T cells (41). The ICI and chemotherapy-bevacizumab regimen and the ICI and chemotherapy with radiation regimen did not significantly differ in efficacy.

Further subgroup analysis revealed insignificant differences between the groups treated with ICI plus chemotherapy with bevacizumab and those without bevacizumab. However, the COMPASSION-16 study confirmed that cadonilimab plus chemotherapy was more superior than the blended treatment of cadonilimab plus chemotherapy plus bevacizumab in recruiting more activated NK cells, although it also resulted in more side effects, such as vaginal bleeding, leg edema, and palpitations.

Cadonilimab might provide a better option for patients who cannot tolerate bevacizumab (3), as indicated by these findings. Despite preclinical data suggesting a synergistic benefit between immunotherapy and bevacizumab, more validation is required to identify the appropriate combination regimen and ICI type pairing in clinical studies.

### 4.2 The limits of the meta-analysis and systematic review

This systematic review has several inherent limitations. First, the included studies were not directly comparable, and some had small sample sizes, which may have compromised the accuracy of the outcomes when comparing different groups. Second, the types and dosages of immune checkpoint inhibitors varied across trials, potentially introducing bias. Consequently, care should be used when interpreting the findings about the safety and effectiveness of immunological combination treatments for ACC. To accurately evaluate the effect of immunological combination therapy on safety and efficacy in patients with advanced tumor, large, global, randomized double-blind controlled trials with prolonged follow-up are required.

## 5 Conclusion

Based on the meta-analysis comparing combined immunotherapy with non-immune therapy, combination immunotherapy significantly improved OS, PFS, ORR, and DCR. When compared to monotherapy, dual ICI therapy did not significantly alter safety or efficacy during long-term follow-up. Randomized controlled trials should be conducted to further validate its potential value in overcoming resistance. Subgroup analysis indicated that patients under 65 years old or with squamous cell carcinoma subtype gained more benefit from combined immunotherapy. Bevacizumab inclusion or exclusion should be determined on an individual basis.

Studies on patient tolerance and treatment strategies in combination therapy show a slight increase in TRAEs. Maintaining patients’ quality of life requires careful observation and individualized care. Future research should build thorough toxicity management protocols, identify patient subgroups that benefit most from particular treatments, and ascertain whether the existing regimen is ideal for all cases.

## Data Availability

The original contributions presented in the study are included in the article/**Supplementary Material**. Further inquiries can be directed to the corresponding authors.
