# Supplementary material for: Efficacy and safety of combined immune therapy for advanced cervical cancer: a systematic review and meta-analysis
Source: Front Oncol. 2026 May 25;16:1772054. doi: 10.3389/fonc.2026.1772054 (PMC13243269; doi:10.3389/fonc.2026.1772054)
Supplement: Supplementary file 1 [file Table1.docx]

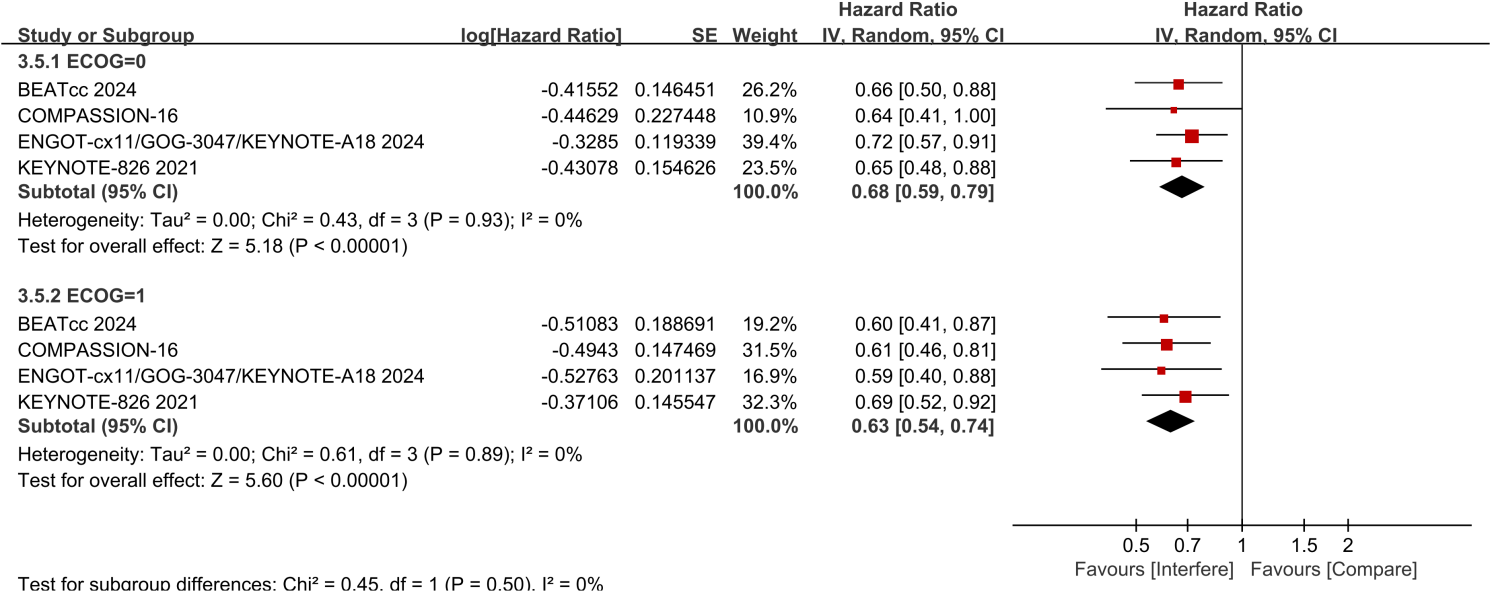


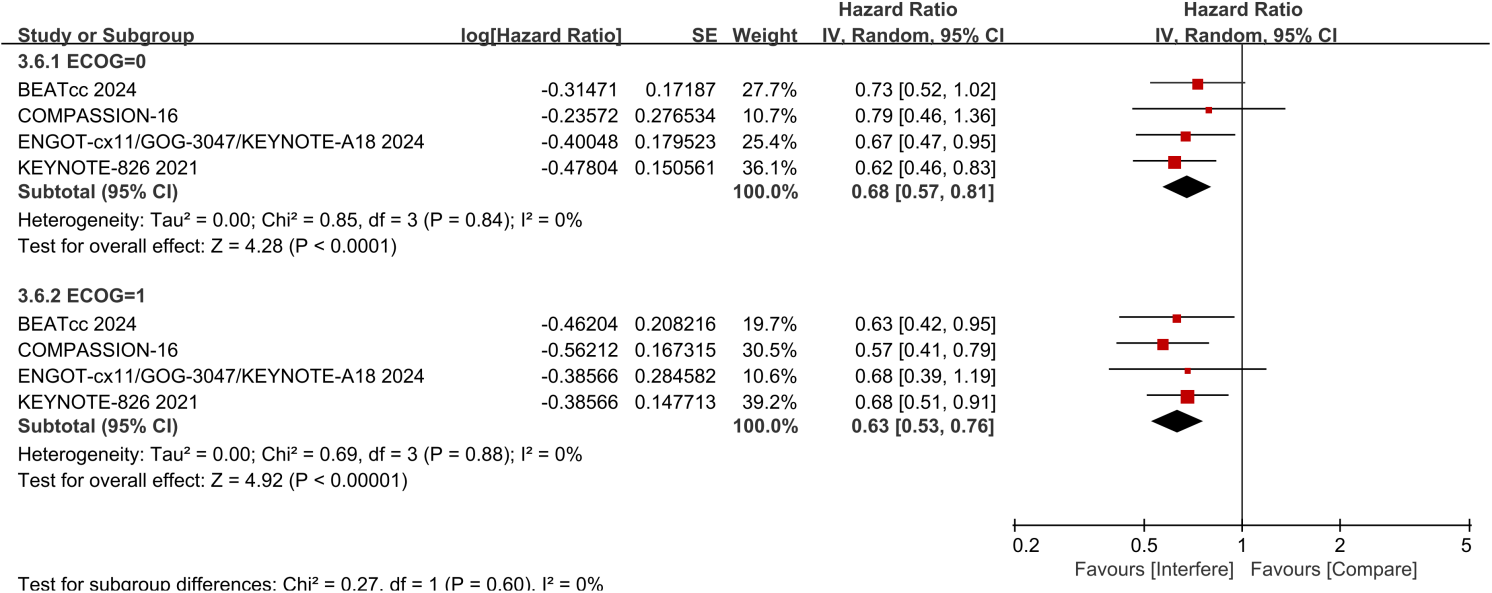


**Supplementary Figure S1. ECOG (PFS and OS)**


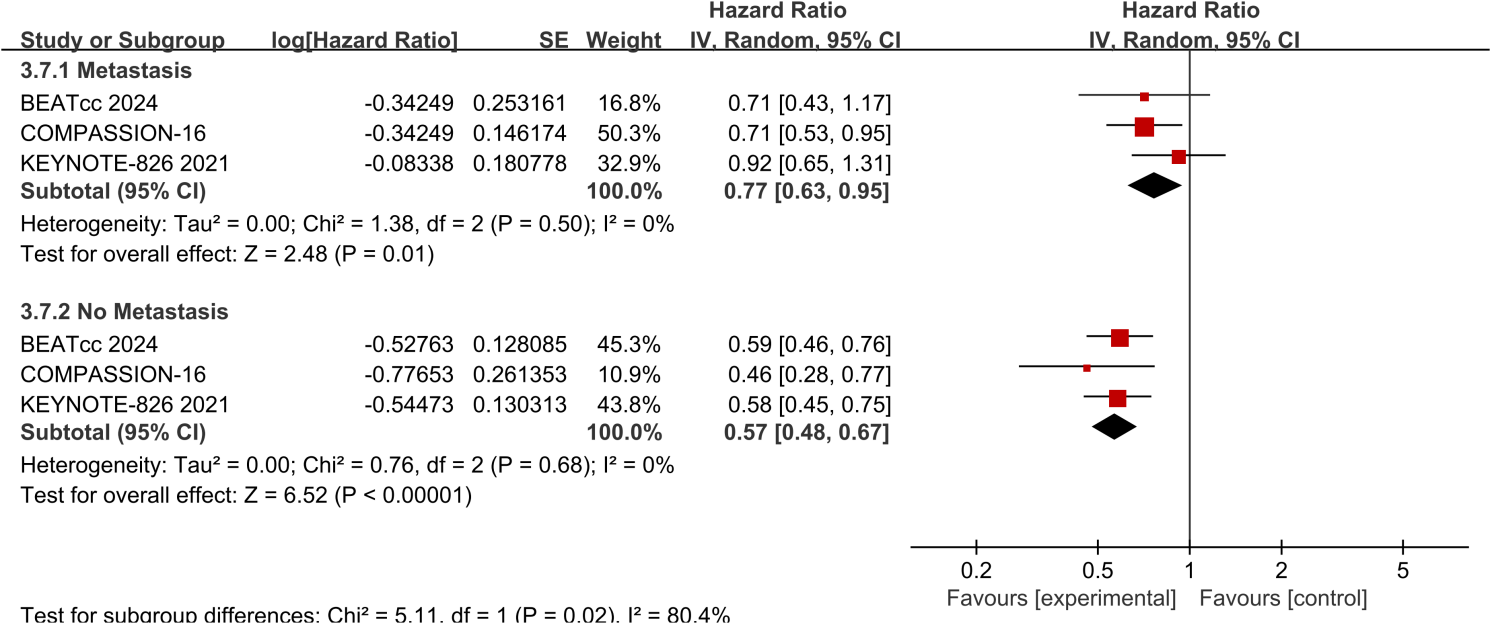


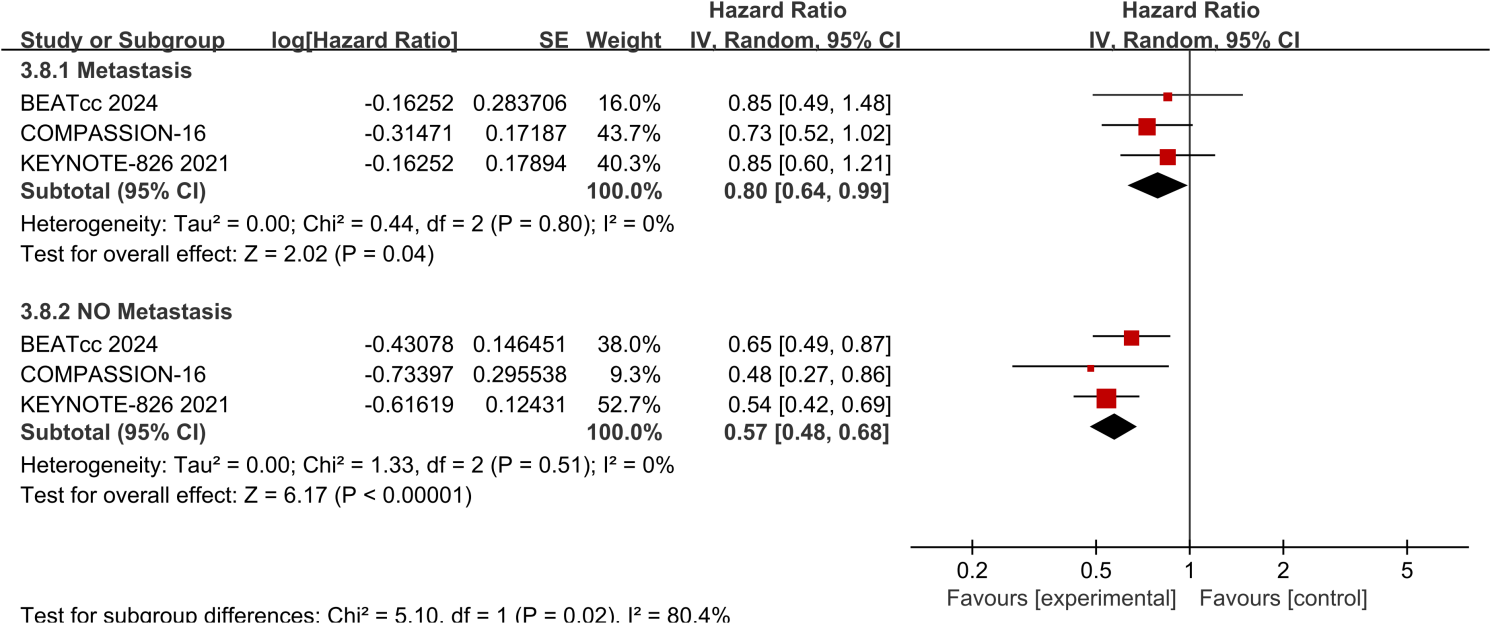


**Supplementary Figure S2.** **Metastasis or No Metastasis (PFS and OS)**


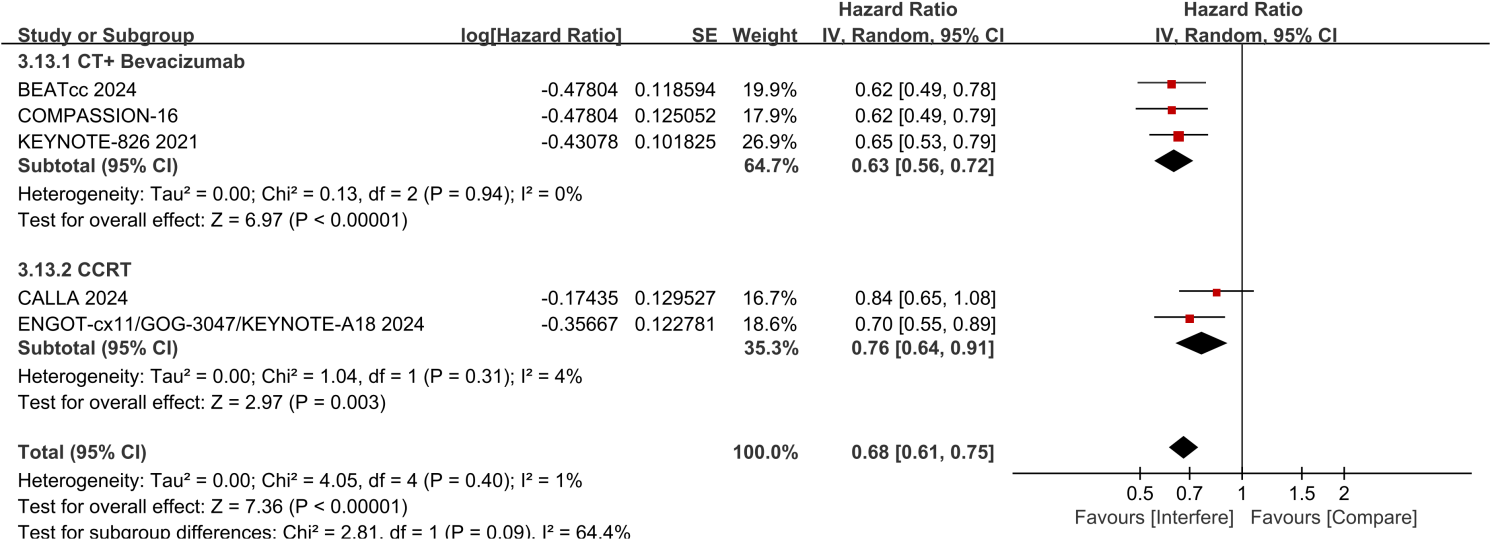


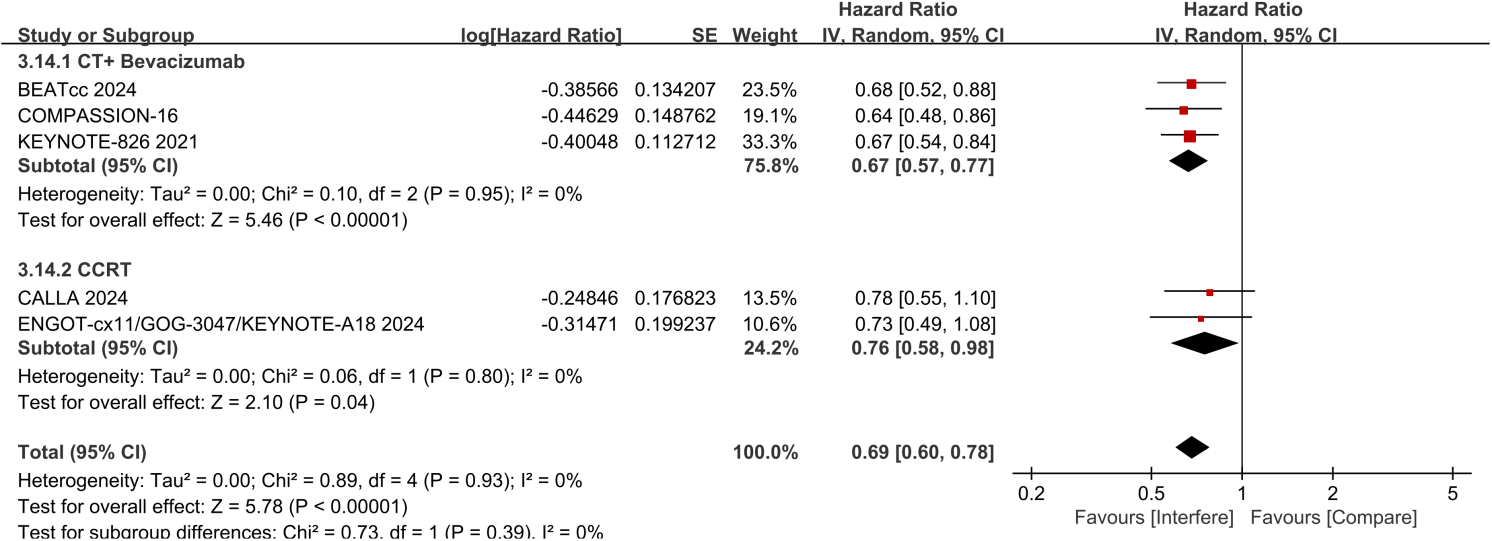


**Supplementary Figure S3.** **Treatment Regimen (PFS and OS)**

**
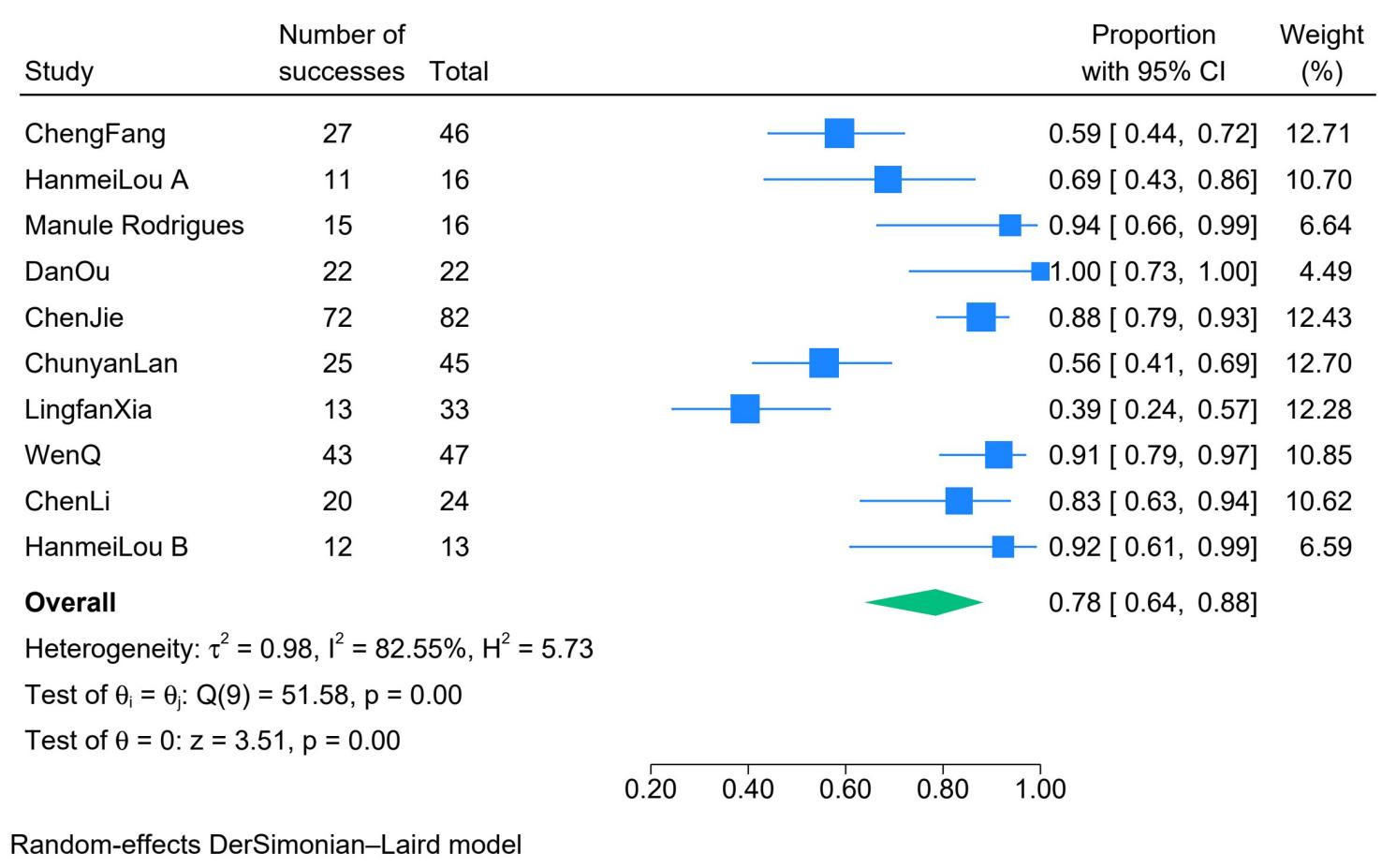
**

**Supplementary Figure S4. Forest plot of objective response rate (ORR) in single-arm studies**

**
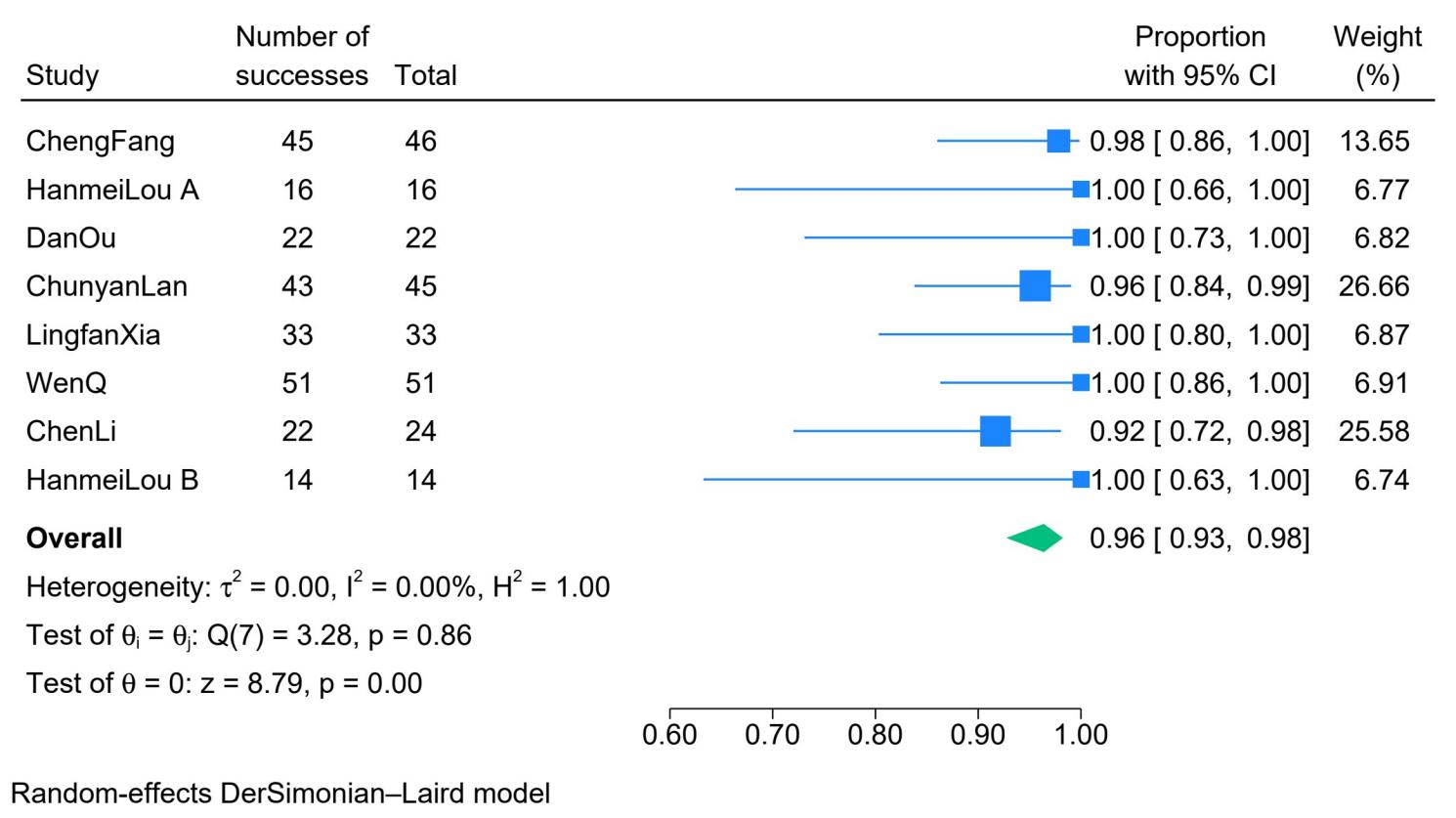
**

**Supplementary Figure S5. Forest plot of the incidence of treatment-related adverse events in single-arm studies**

**
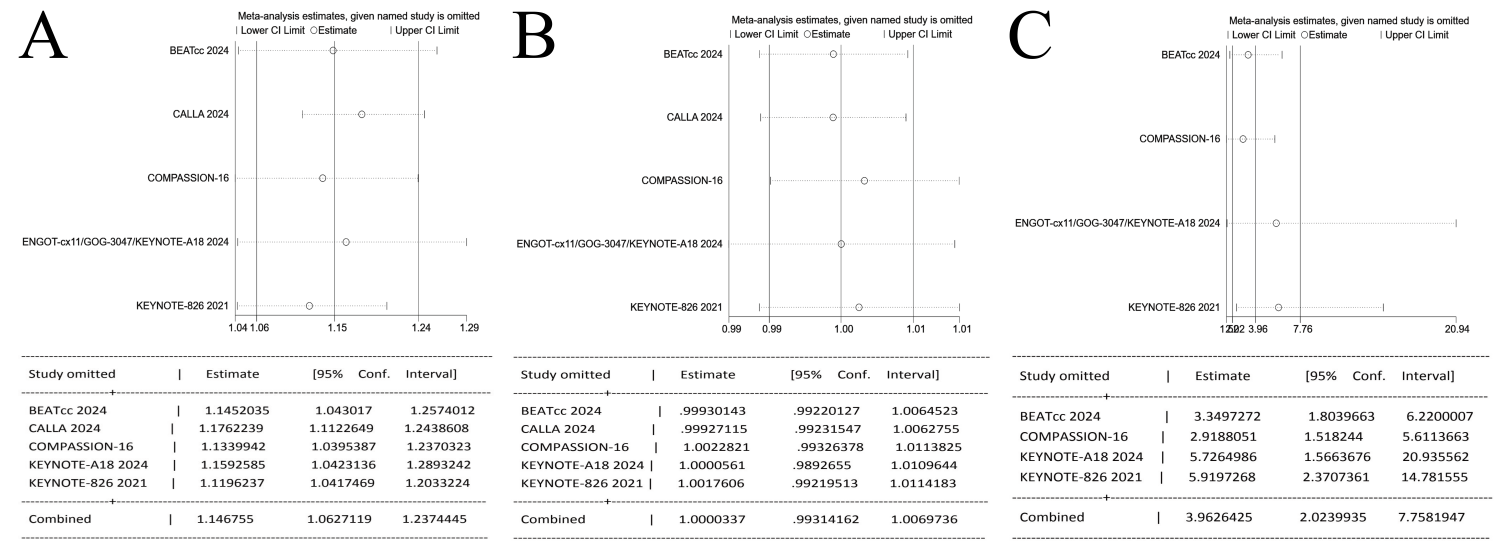
**

**Supplementary Figure S6. Sensitivity analyses for ORR (A), TRAEs (B), and irAEs (C) conducted using the leave-one-out approach**

**ORR, objective response rate; TRAEs, treatment-related adverse events; irAEs, immune-related adverse events.**

**
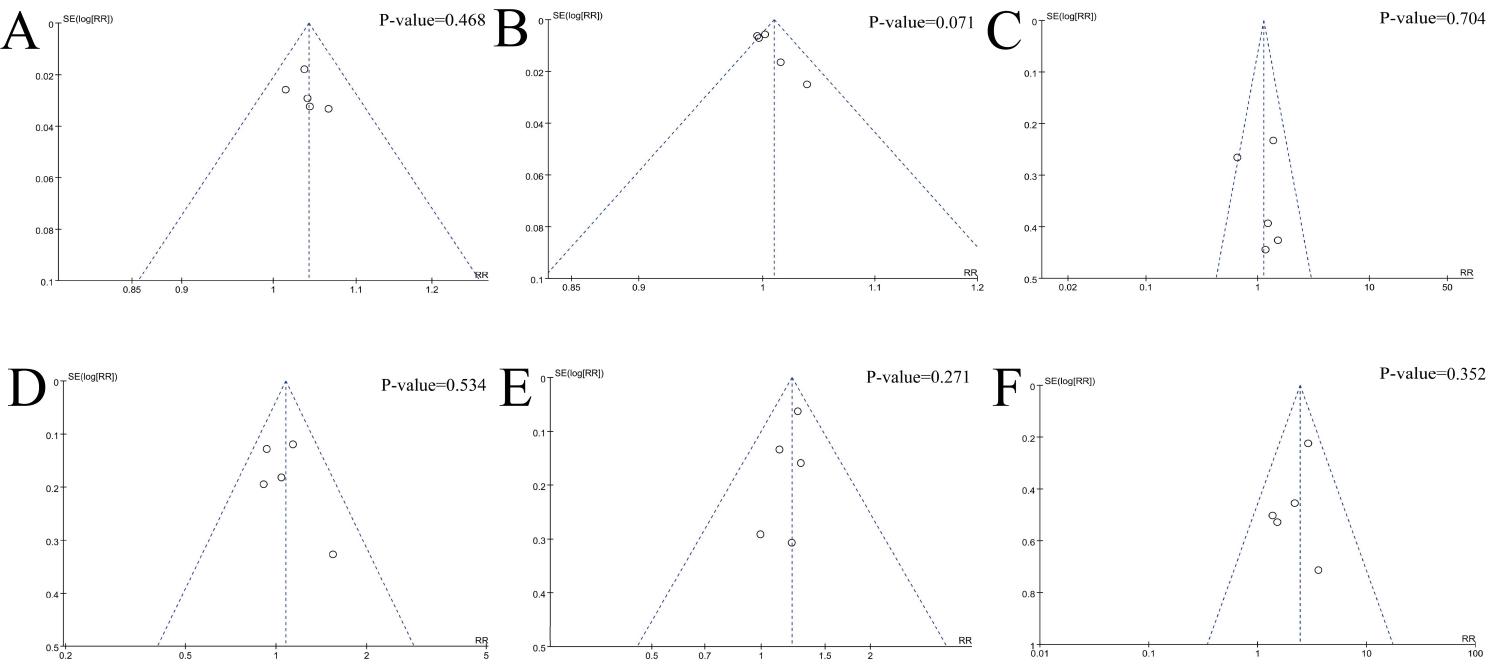
**

**Supplementary Figure S7. Funnel plots and Egger’s regression tests**

**In immune combination therapy: (A) DCR, (B) TRAEs.**

**In dual immune checkpoint inhibitor (ICI) therapy: (C) ORR, (D) DCR, (E) TRAEs, (F) grade 3-5 TRAEs.**

**DCR, disease control rate; TRAEs, treatment-related adverse events; ORR, objective response rate.**

**
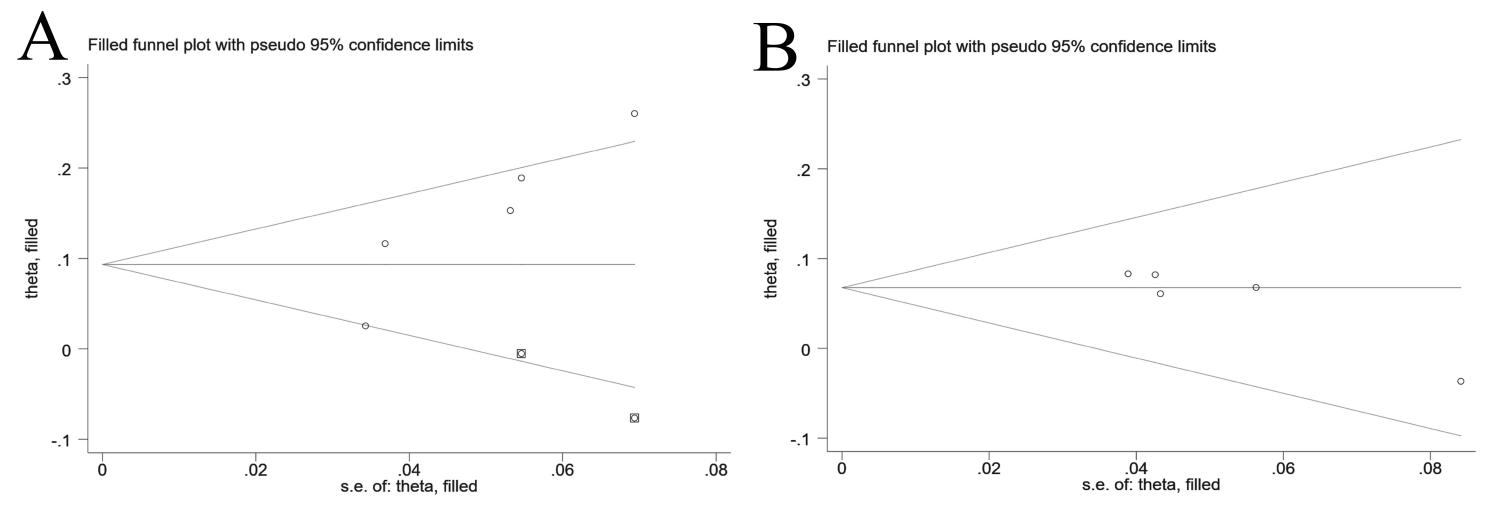
**

**Supplementary Figure S8. Funnel plots with Trim and Fill method for randomized controlled trials of immune combination therapy (A) ORR; (B) G3-5 TRAEs**

**ORR, objective response rate; TRAEs, treatment-related adverse events.**


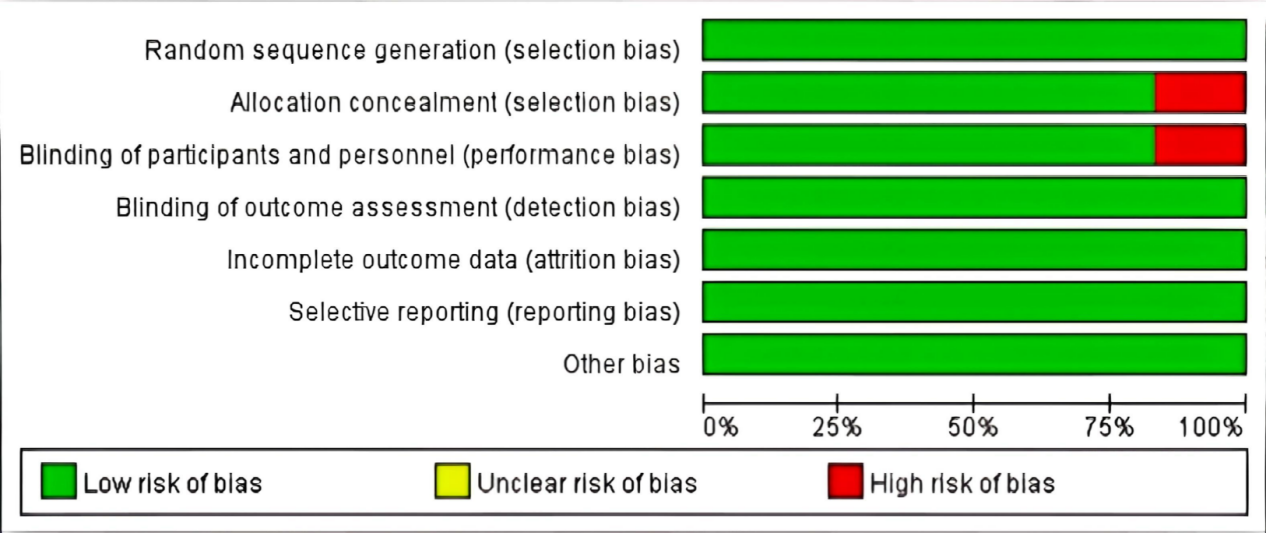

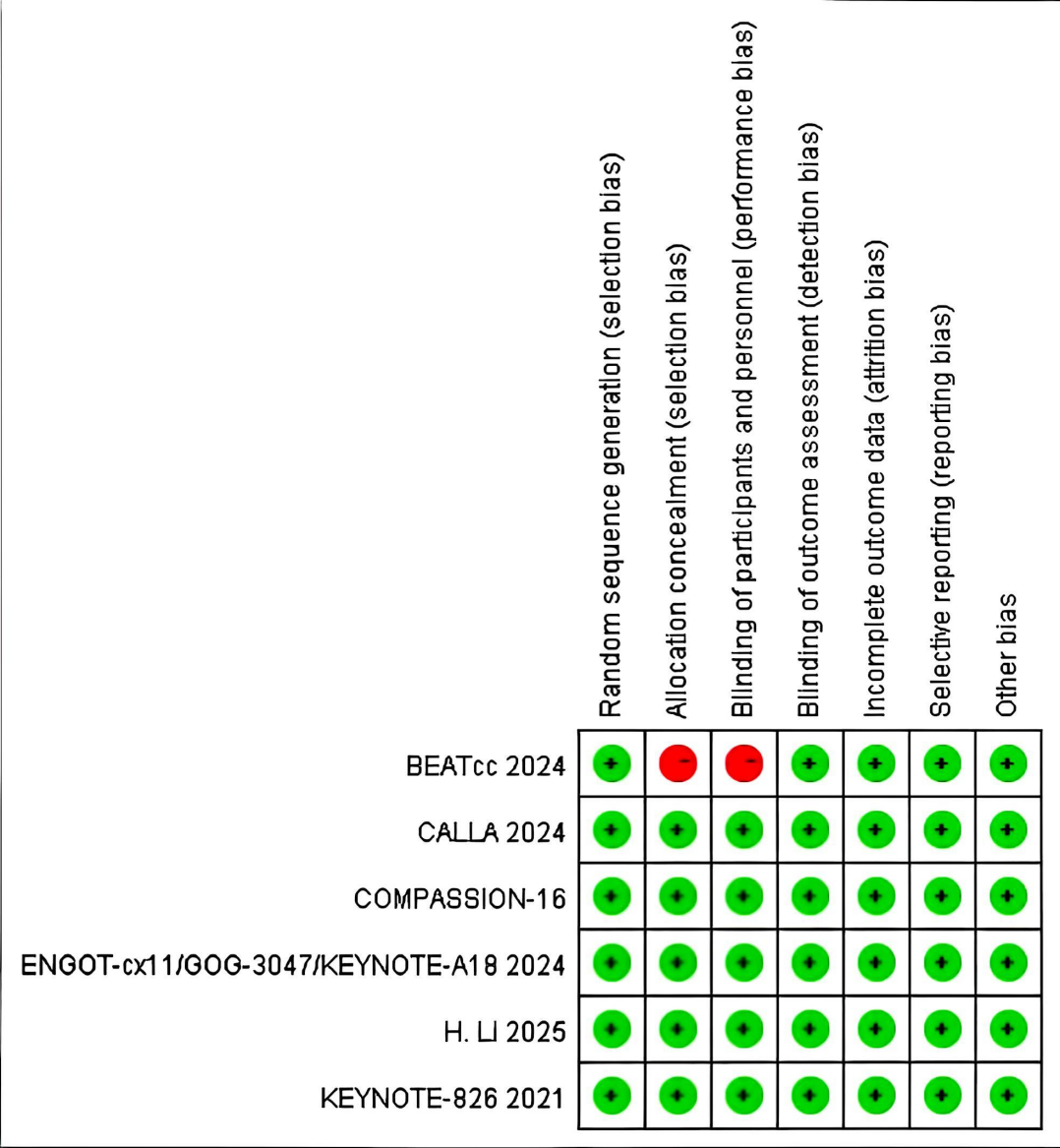


**Supplementary Figure S9. Risk of bias assessment：RoB-2 Assessment Form**

**Supplementary Table S1. Search term**

| **Pubmed** |  |  |
| --- | --- | --- |
| #1 | (("Uterine Cervical Neoplasms"[Mesh]) OR (((((((((((((((((Uterine Cervical Neoplasm[Title/Abstract]) OR (Cervical Neoplasm*, Uterine[Title/Abstract])) OR (Neoplasm*, Uterine Cervical[Title/Abstract])) OR (Neoplasm*, Cervical[Title/Abstract])) OR (Cervical Neoplasm*[Title/Abstract])) OR (Neoplasm*, Cervix[Title/Abstract])) OR (Cervix Neoplasm*[Title/Abstract])) OR (Cancer of the Uterine Cervix[Title/Abstract])) OR (Cancer of the Cervix[Title/Abstract])) OR (Cervical Cancer*[Title/Abstract])) OR (Cancer*, Cervical[Title/Abstract])) OR (Cancer*, Uterine Cervical[Title/Abstract])) OR (Cervical Cancer*, Uterine[Title/Abstract])) OR (Uterine Cervical Cancer*[Title/Abstract])) OR (Cancer of Cervix[Title/Abstract])) OR (Cervix Cancer*[Title/Abstract])) OR (Cancer*, Cervix[Title/Abstract]))) | 117,003 |
| #2 | (("Immune Checkpoint Inhibitors"[Mesh]) OR (((((((((((((((((((((((((((((((((((((((ICIs[Title/Abstract]) OR (Immune Checkpoint Inhibitor[Title/Abstract])) OR (Checkpoint Inhibitor*, Immune[Title/Abstract])) OR (Immune Checkpoint Blocker*[Title/Abstract])) OR (Checkpoint Blocker*, Immune[Title/Abstract])) OR (Immune Checkpoint Blockade[Title/Abstract])) OR (Checkpoint Blockade ,Immune[Title/Abstract])) OR (Immune Checkpoint Inhibition[Title/Abstract])) OR (Checkpoint inhibition ,immune[Title/Abstract])) OR (PD-L1 Inhibitor*[Title/Abstract])) OR (PD-1 Inhibitor*[Title/Abstract])) OR (PD L1 Inhibitor*[Title/Abstract])) OR (PD 1 Inhibitor*[Title/Abstract])) OR (Inhibitor*, PD-1[Title/Abstract])) OR (Programmed Death-Ligand 1 Inhibitor*[Title/Abstract])) OR (Programmed Death Ligand 1 Inhibitor*[Title/Abstract])) OR (Programmed Cell Death Protein 1 Inhibitor*[Title/Abstract])) OR (PD-1-PD-L1 Blockade[Title/Abstract])) OR (Blockade,PD-1-PD-L1[Title/Abstract])) OR (PD 1 PD L1 Blockade[Title/Abstract])) OR (CTLA-4 Inhibitor*[Title/Abstract])) OR (CTLA 4 Inhibitor*[Title/Abstract])) OR (Cytotoxic T Lymphocyte Associated Protein 4 Inhibitor*[Title/Abstract])) OR (Cytotoxic T -Lymphocyte-Associated Protein 4 Inhibitor*[Title/Abstract])) OR (Pembrolizumab[Title/Abstract])) OR (Nivolumab[Title/Abstract])) OR (Atezolizumab[Title/Abstract])) OR (Durvalumab[Title/Abstract])) OR (Cemiplimab[Title/Abstract])) OR (Camrelizumab[Title/Abstract])) OR (Sintilimab[Title/Abstract])) OR (Tislelizumab[Title/Abstract])) OR (Toripalimab[Title/Abstract])) OR (Avelumab[Title/Abstract])) OR (Tremelimumab[Title/Abstract])) OR (Ipilimumab[Title/Abstract])) OR (Dostarlimab[Title/Abstract])) OR (Balstilimab[Title/Abstract])) OR (Camrelizumab[Title/Abstract]))) | 60,788 |
| #3 | #1 AND #2 | 633 |
| Embase |  |  |
| #1 | uterine cervix tumor'/exp OR 'cervical neoplasia':ab,ti OR 'cervical neoplasm':ab,ti OR 'cervical tumor':ab,ti OR 'cervical tumorigenesis':ab,ti OR 'cervical tumour':ab,ti OR 'cervix neoplasia':ab,ti OR 'cervix neoplasm':ab,ti OR 'cervix neoplasms':ab,ti OR 'cervix tumor':ab,ti OR 'cervix tumorigenesis':ab,ti OR 'cervix tumour':ab,ti OR 'cervix uteri tumor':ab,ti OR 'neoplasia of the cervix':ab,ti OR 'neoplasm of the cervix':ab,ti OR 'neoplastic cervical':ab,ti OR 'neoplastic cervix':ab,ti OR 'tumor of the cervix':ab,ti OR 'tumor of the uterine cervix':ab,ti OR 'tumour of the cervix':ab,ti OR 'tumour of the uterine cervix':ab,ti OR 'uterine cervical neoplasia':ab,ti OR 'uterine cervical neoplasm':ab,ti OR 'uterine cervical neoplasms':ab,ti OR 'uterine cervical tumor':ab,ti OR 'uterine cervix neoplasia':ab,ti OR 'uterine cervix neoplasm':ab,ti OR 'uterine cervix tumour':ab,ti OR 'uterine cervix tumor':ab,ti | 169933 |
| #2 | immune checkpoint inhibitor'/exp OR 'immune checkpoint blocker':ab,ti OR 'immune checkpoint inhibitor':ab,ti OR 'pd 1 inhibitor':ab,ti OR 'immune checkpoint blockade':ab,ti OR 'immune checkpoint inhibition':ab,ti OR 'pd l1 inhibitor':ab,ti OR 'programmed death ligand 1 inhibitor':ab,ti OR 'programmed cell death protein 1 inhibitor':ab,ti OR pembrolizumab:ab,ti OR nivolumab:ab,ti OR atezolizumab:ab,ti OR durvalumab:ab,ti OR cemiplimab:ab,ti OR ipilimumab:ab,ti OR camrelizumab:ab,ti | 120273 |
| #3 | #1 AND #2 | 1997 |
| Cochrane |  |  |
| #1 | MeSH descriptor: [Immune Checkpoint Inhibitors] explode TI trees or PD L1 Inhibitors or PD L1 Inhibitor or Programmed Death-Ligand 1 Inhibitors or Programmed Death Ligand 1 Inhibitors or PD 1 PD L1 Blockade or Immune Checkpoint Blockers or Immune Checkpoint Inhibitor or Immune Checkpoint Inhibitors or CTLA-4 Inhibitor or Cytotoxic T-Lymphocyte-Associated Protein 4 Inhibitor or Cytotoxic T-Lymphocyte-Associated Protein 4 Inhibitors or CTLA-4 Inhibitors or Cytotoxic T Lymphocyte Associated Protein 4 Inhibitor or Cytotoxic T Lymphocyte Associated Protein 4 Inhibitors or PD 1 Inhibitors or PD 1 Inhibitor or Programmed Cell Death Protein 1 Inhibitor or Programmed Cell Death Protein 1 Inhibitors or Immune Checkpoint Inhibition or Immune Checkpoint Blockade | 11882 |
| #2 | MeSH descriptor: [Uterine Cervical Neoplasms] explode TI trees or Neoplasm or Uterine Cervical or Cervical Neoplasm, Uterine or Uterine Cervical Neoplasm or Cervix Neoplasms or Neoplasms, Cervical or Cervix Neoplasm or Neoplasm, Cervix or Neoplasms, Cervix or Cervical Neoplasms or Cervical Neoplasm or Cervical Cancers or Cancer of Cervix or Uterine Cervical Cancer or Cervix Cancer or Cancer, Cervix or Cancer, Uterine Cervical or Uterine Cervical Cancers or Cancer of the Uterine Cervix or Cervical Cancer or Cervical Cancer, Uterine or Cancer, Cervical or Cancer of the Cervix | 50835 |
| #3 | #1 AND #2 | 1097 |
| WOS |  |  |
| #1 | TS=(Uterine Cervical Neoplasms OR Uterine Cervical Neoplasm OR Cervical Neoplasm*, Uterine OR Neoplasm*, Cervical OR Cervical Neoplasm* OR Neoplasm*, Cervix OR Cervix Neoplasm* OR Cancer of the Uterine Cervix OR Cancer of Cervix OR cervical cancer* OR Cancer*, Cervical OR cervical carcinoma OR Uterine Cervical Cancer* OR Cancer*, Uterine Cervical OR cervical cancer*, Uterine OR Cervix Cancer* OR Cancer of the Cervix OR carcinoma of cervix OR Cancer*, Cervix) | 288,281 |
| #2 | TS=( Immune Checkpoint Inhibitors OR ICIs OR Immune Checkpoint Inhibitor OR Checkpoint Inhibitor*, Immune OR Immune Checkpoint Blocker* OR Checkpoint Blocker*, Immune OR Immune Checkpoint Blockade OR Checkpoint Blockade, Immune OR Immune Checkpoint Inhibition OR Checkpoint Inhibition, immune OR PD-L1 Inhibitor* OR PD-1 Inhibitor* OR PD L1 Inhibitor* OR PD 1 Inhibitor* OR Inhibitor*, PD-1 OR Programmed Death-Ligand 1 Inhibitor* OR Programmed Death Ligand 1 Inhibitor* OR Programmed Cell Death Protein 1 Inhibitor* OR PD-1-PD-L1 Blockade OR Blockade, PD-1-PD-L1 OR PD 1 PD L1 Blockade OR CTLA-4 Inhibitor* OR CTLA 4 Inhibitor* OR Cytotoxic T Lymphocyte Associated Protein 4 Inhibitor* OR Cytotoxic T -Lymphocyte-Associated Protein 4 Inhibitor* OR Pembrolizumab OR Nivolumab OR Atezolizumab OR Camrelizumab OR Sintilimab OR Tislelizumab OR Toripalimab OR Ipilimumab OR Dostarlimab OR Balstilimab OR Camrelizumab OR Durvalumab OR Cemiplimab OR Avelumab OR Tremelimumab) | 166,244 |
| #5 | #1 AND #2 | 2,238 |

**Supplementary Table S2. Main characteristic of study of ICIs in advanced CC**

| **Study Name** | **Author, year** | **country** | **recruitment** | **design(Phase)** | follow-up | **sample size** | **age** | **cancer status** | **interfere/control** | **The expression of PD-L1(%)** | **Histological Subtype(n/N)** | **Administered Drugs** |
| --- | --- | --- | --- | --- | --- | --- | --- | --- | --- | --- | --- | --- |
|  |  |  |  |  |  |  |  |  |  |  |  |  |
| CALLA | AstraZeneca , et al.,2024 | American | 2019-2022 | III(RCT) | the durvalumab group : Median follow-up was 18·5 months (IQR 13·2-21·5) | Durvalumab group：n=385 | Age ≥18 | Documented evidence of cervical adenocarcinoma or squamous carcinoma FIGO (2009) Stages IB2 to IIB node positive or FIGO (2009) IIIA-IVA any node | **Interfere** **:** durvalumab + standard of care concurrent chemoradiation therapy (SoC CCRT) followed by durvalumab monotherapy up to 24 months or until PD from the date of randomization | Durvalumab group: evaluated 371 people PD-L1≥1% n=356 ； PD-L1>5% n=311. | Squamous carcinoma：n=322/385; Adenocarcinoma: n=55/385; Adenosquamous : n=8/385 | Durvalumab 1500mg IV infusion every 4 weeks plus Standard of Care (SoC) concurrent chemoradiotherapy (CCRT) (chemotherapy for 5 weeks plus external beam radiotherapy and brachytherapy) |
|  |  |  |  |  | the placebo group : Median follow-up was 18·4 months (IQR 13·2-23·7) | Control therapy : n=385 |  |  | **Control :** placebo + standard of care concurrent chemoradiation therapy (SoC CCRT) | Control therapy group: evaluated 364 people PD-L1≥1% n=352 PD-L1>5% n=300 | Squamous carcinoma：n=320/385; Adenocarcinoma: n=58/385; Adenosquamous : n=7/385 | Placebo IV infusion every 4 weeks plus Standard of Care (SoC) concurrent chemoradiotherapy (CCRT) (chemotherapy for 5 weeks plus external beam radiotherapy and brachytherapy) |
| （ENGOT-cx11/GOG-3047/KEYNOTE-A18） | Tewari, et al.,2024 | American | 2020-2022 | III（RCT） | Median follow-up was 17.9 months (IQR11.3-22.3) | pembrolizumab group: n=529 | Age≥18 | newly diagnosed, high-risk, locally advance cervical cancer | **Interfere** : pembrolizumab + CCRT | pembrolizumab group: evaluated 529 people PD-L1≥1% n=502，PD-L <1% n=22. | Non-squamous：n=96/529；Squamous：n=433/529 | 5 cycles of pembrolizumab 200 mg every  3 weeks plus chemoradiotherapy, followed by 15 cycles of  pembrolizumab 400 mg every 6 weeks. |
|  |  |  |  |  |  | Control therapy: n=531 |  |  | **Control** : placebo + CCRT | Control therapy: evaluated 531 people PD-L1 ≥1%: n=498 ; PD-L1 <1%:n=28 | Non-squamous：n=80/529；Squamous：n=451/529 | 5 cycles of placebo every 3 weeks plus chemoradiotherapy, followed by 15 cycles of placebo every 6 weeks |
| KEYNOTE-826 | Nicoletta Colombo et al., 2021 | Italy | 2018-2020 | III（RCT） | the time from randomization to the May 3, 2021, data cutoff for the first interim analysis, was 22.0 months (range, 15.1 to 29.4) | pembrolizumab group: n=308 | Age ≥18 | persistent, recurrent, or metastatic adenocarcinoma, adenosquamous carcinoma, or squamous-cell carcinoma of the cervix | **Interfere:** pembrolizumab+platinum-based chemotherapy+bevacizumab | pembrolizumab group: evaluated 308 people PD-L<1% n=35 ； PD-L1 1-10% n=115 , PD-L1 ≥10% n=158 | Adenocarcinoma: 56/308 ; Squamous cell carcinoma: 235/308 ; Adenosquamous carcinoma: 15/308 | pembrolizumab (200 mg) every 3 weeks for up to 35 cycles. The patients were to receive paclitaxel (175 mg per square meter of body-surface area) and the investigator’s choice of cisplatin (50 mg per square meter) or carboplatin (area under the concentration–time curve, 5 mg per milliliter per minute) every 3 weeks. |
|  |  |  |  |  |  | placebo group: n=309 |  |  | **Control:**placebo+platinum-based chemotherapy+bevacizumab | Control therapy group: evaluated 309 people PD-L<1% n=34 ； PD-L1 1-10% n=116 , PD-L1 ≥10% n=159 | Adenocarcinoma: 84/309 ; Squamous cell carcinoma: 211/309 ; Adenosquamous carcinoma: 14/309 | placebo (200mg) every 3 weeks for up to 35 cycles.The patients were to receive paclitaxel (175 mg per square meter of body-surface area) and the investigator’s choice of cisplatin (50 mg per square meter) or carboplatin (area under the concentration–time curve, 5 mg per milliliter per minute) every 3 weeks. |
| BEATcc | Ana Oaknin et al., 2024 | Spain | 2018-2021 | III（RCT） | median duration of follow-up was 32·9 months (95% CI 31·2–34·6) | atezolizumab group: n=206 | Age ≥18 | (according to Response Evaluation Criteria in Solid Tumours version 1.1 [RECIST]) metastatic (stage IVB), persistent, or recurrent cervical cancer | **Interfere:**bevacizumab + chemotherapy+atezolizumab | Not mentioned | Adenocarcinoma: n=36/206(17%) Squamous-cell carcinoma: n=164/206(80%) Adenosquamous cell carcinoma: n=6/206(3%） | intravenous platinum (cisplatin 50 mg/m² or carboplatin area under the curve of 5), intravenous paclitaxel 175 mg/m², and intravenous bevacizumab 15 mg/kg, all on day 1 of every 3-week cycle and atezolizumab 1200 mg on day 1 of every 3-week cycle |
|  |  |  |  |  |  | placebo group: n=204 |  |  | **Control:**bevacizumab + chemotherapy | Not mentioned | Adenocarcinoma: n=43/204(21%) Squamous-cell carcinoma: n=157/204(77%) Adenosquamous cell carcinoma: n=4/204(2%） | intravenous platinum (cisplatin 50 mg/m² or carboplatin area under the curve of 5), intravenous paclitaxel 175 mg/m², and intravenous bevacizumab 15 mg/kg, all on day 1 of every 3-week cycle |
| COMPASSION-16 | Xiaohua Wu et al.,2024 | China | 2021-2022 | III（RCT） | the median follow-up was  25·6 months (IQR 23·6–28·0) | Cadonilimab group: n=222 | Age (18-75) | (stage IVB) cervical cancer of squamous cell carcinoma, adenocarcinoma, or adenosquamous carcinoma | **Interfere:** Cadonilimab + platinum-based chemotherapy + bevacizumab | Durvalumab group: evaluated 222 people PD-L1<1% n=62/222 ； PD-L1 1 to <10% n=64/222 ; PD-L1<10% n=91/222 | Squamous cell carcinoma: 182/222; Adenocarcinoma: 34/222; Adenosquamous carcinoma: 6/222 | cadonilimab (10 mg/kg) in combination with chemotherapy (cisplatin [50 mg/m²] or carboplatin [area under the curve 4–5] plus paclitaxel [175 mg/m²]), with or without bevacizumab (15 mg/kg). |
|  |  |  |  |  |  | placebo group: n=223 |  |  | **Control:** platinum-based chemotherapy + bevacizumab | Control therapy group: evaluated 309 people PD-L<1% n=54/223； PD-L1 1-10% n= 68/223, PD-L1 ≥10% n= 89/223 | Squamous-cell carcinoma: n=188/223;Adenocarcinoma: n=29/223; Adenosquamous carcinoma: 6/223 | placebo(10 mg/kg) in combination with chemotherapy (cisplatin [50 mg/m²] or carboplatin [area under the curve 4–5] plus paclitaxel [175 mg/m²]), with or without bevacizumab (15 mg/kg). |
| SKYSCRAPER-04 | Hoffmann-La Roche, et al.,2025 | Swiss Confederation | 2020-2025 | II | Not mentioned | Tiragolumab plus Atezolizumab group : n=126 | Age  ≥ 18 | recurrent or persistent squamous cell carcinoma, adenosquamous carcinoma, or adenocarcinoma of the cervix | **Interfere**:Tiragolumab + Atezolizumab | Tiragolumab plus Atezolizumab group : |  | Tiragolumab at a fixed dose of 600 milligrams (mg) will be administered by intravenous (IV) infusion every 3 weeks (Q3W) on Day 1 of each 21-day cycle + Atezolizumab at a fixed dose of 1200 mg will be administered by IV infusion Q3W on Day 1 of each 21-day cycle. |
|  |  |  |  |  |  | Atezolizumab group : n=45 |  |  | **Control**: Atezolizumab | Atezolizumab group : |  | Atezolizumab at a fixed dose of 1200 mg will be administered by IV infusion Q3W on Day 1 of each 21-day cycle. |
| IBI310 plus sintilimab vs. placebo plus sintilimab in recurrent/metastatic cervical cancer: A doubleblind, randomized controlled trial | Huayi Li , et al.,2025 | China | 2020-2022 | II(RCT) | the median follow-up times were 13.0 months (95% confidence interval [CI]: 12.5–14.3) | IBI310 plus sintilimab group (n = 103) | Median (IQR) 53 (47–58), <=65 years (%) 95 (93) , >65 years (%) 7 (7). | recurrent/metastatic cervical cancer | **Interfere:** IBI310 plus sintilimab | IBI310 plus sintilimab group: evaluated 222 people PD-L1≥1% n=71/102 ; PD-L1 1 to <1% n=31/102 | Squamous-cell carcinoma: n=86/102;Adenocarcinoma: n=16/102; Adenosquamous carcinoma: n=0/102. | IBI310 3mg/kg + sintilimab 200mg |
|  |  |  |  |  | the median follow-up times were 12.8 months (95% CI: 11.7–14.5) | placebo plus sintilimab (n = 102) | Median (IQR) 53 (47–59) <=65 years (%) 93 (92) >65 years (%) 8 (8) |  | **Control:** placebo plus sintilimab | Control therapy group: evaluated 101 people PD-L1 ≥1% n=72/101； PD-L1 <1% n= 29/101 | Squamous-cell carcinoma: n=91/101;Adenocarcinoma: n=7/101; Adenosquamous carcinoma: n=3/101. | Placebo + sintilimab 200mg |
| AdvanTIG-202 | J-Y.Lee ，et al.,2023 | Korea | As of June 16, 2022 | II(RCT) | median study follow-up: 7.4 mos | TIS + OCI group : n=138 | median age was 53.0 years (IQR 46.0–59.0) | recurrent/metastatic (R/M) cervical cancer (CC) | **Interfere:**  TIS + OCI | TIS + OCI group: evaluated 222 people PD-L1≥5% n=84/138 ; PD-L1 1 to <5% n=53/138 | Squamous-cell carcinoma: n=107/138;Adenocarcinoma: n=27/138; Adenosquamous carcinoma: n=4/138. | 200 mg TIS IV Q3W + 900 mg OCI IV Q3W |
|  |  |  |  |  |  | TIS monotherapy group : n=40 | median age was 51.0 years (IQR 47.0–59.0) |  | **Control:** TIS | TIS group: evaluated 101 people PD-L1 ≥5% n=20/40； PD-L1 <5% n= 20/40 | Squamous-cell carcinoma: n=29/40;Adenocarcinoma: n=11/40; Adenosquamous carcinoma: n=0/40. | 200 mg TIS IV Q3W |
| QL1604 plus paclitaxel-cisplatin/carboplatin in patients with recurrent or metastatic cervical cancer: an open-label, single-arm, phase II trial | Cheng Fang ,et al.,2024 | China | 2020-2021 | II(single arm) | the median follow-up duration was 16.5 months | QL1604 plus paclitaxel-cisplatin/carboplatin :n=46 | Age (18-75) | recurrent or metastatic cervical cancer | QL1604 + paclitaxel-cisplatin/carboplatin | PD-L1 CPS<1 n=5 (10.9%) CPS: 1–49 n=38 (82.6%) CPS ≥ 50 n=3 (6.5%) | Squamous-cell carcinoma: n=40/46;Adenocarcinoma: n=5/46; Adenosquamous carcinoma: n=1/46. | QL1604 200 mg, paclitaxel 175 mg/m2, and cisplatin 70 mg/m2 or carboplatin AUC 6 intravenously once Q3W in 3-week cycles for up to 6 cycles |
| COMPASSION-13 | Hanmei Lou ,et al.,2024 | China | 2021 .4 .26 - 2021 .11 . 5 | Cohort study | cohort A-10 : the median duration of follow-up was 18.30 months (95% CI, 15.9–19.4) | cohort A-10 : n=15 | Age (18-75) | recurrent or metastatic cervical cancer with squamous cell, adenocarcinoma, or adenosquamous histology | cohort A-10 : cadonilimab 15 mg/kg every 3 weeks+chemotherapy | PD-L1 CPS <1 n=8 (50.0%)  CPS 1-10 n=4 (25.0%)  CPS ≥10 n=4 (25.0%) | Adenocarcinoma: n=1 Squamous cell carcinoma: n=15 | cadonilimab 10 mg/kg every 3 weeks (Q3W) plus chemotherapy |
|  |  |  |  |  | cohort A-15 : the median duration of follow-up was 20.24 months (95% CI, 18.7–20.8) | cohort A-15 : n=16 |  |  | cohort A-15 : cadonilimab 10 mg/kg every 3 weeks+chemotherapy | PD-L1 CPS <1 n=5(33.3%) CPS 1-10 n=5(33.3%) CPS ≥10 n=5(33.3%) | Adenocarcinoma: n=3 Squamous cell carcinoma: n=12 | cadonilimb 15 mg/kg Q3W plus chemotherapy |
|  |  |  |  |  | cohort B-10 : the median duration of follow-up was 15.01 months (95% CI, 12.7–17.7) | cohort B-10 : n=14 |  |  | cohort B-10 :cadonilimab 10 mg/kg every 3 weeks+chemotherapy+bevacizumab | PD-L1 CPS <1 n=4 (28.6%) CPS 1-10 n=6 (42.9%) CPS ≥10 n=4 (28.6%) | Adenocarcinoma: n=1 Squamous cell carcinoma: n=13 | cadonilimab 10 mg/kg Q3W plus chemotherapy and bevacizumab |
| NICOL | Manule Rodrigues ,el al.,2023 | France | 2017-2020 | II(single arm) | Median follow-up was 23.8 months (range: 3.9–26.2) | n=16 | average age 47,9 (27–77) | LACC | Nivolumab plus chemoradiotherapy | Not mentioned | Squamous cell carcinomas n=14 (87%)  adenocarcinomas n=2 (12.5%) | nivolumab plus full course radiotherapy and pulse-dose rate brachytherapy |
| TRACE | Dan Ou ,et al.,2024 | China | 2020-2021 | II(single arm) | Median follow-up was 31.8 months (9.5 to 37.8 months) | n=22 | The median age was 55 years (range 42–72 years) | LACC | CCRT plus toripalimab | PD-L1 CPS < 1 n=11 (50%) CPS 1–4 n= 4 (18.2%) CPS ≥ 5 n=7 (31.8%) | Not mentioned | IMRT **+** Concurrent chemotherapy with cisplatin at a dose of 40 mg/m2 via intravenous infusion was administered once weekly for 5 weeks during EBRT. Toripalimab (240 mg via intravenous infusion) was administered on days 1, 22 and 43 |
| A prospective phase Ⅱ clinical trial of toripalimab combined with platinum-based concurrent chemoradiotherapy and consolidation chemotherapy in patients with locally advanced cervical cancer | Chen Jie ,et al.,2024 | China | 2019-2022 | Ⅱ | 随访时间为20.6（14.0，27.9）个月 | n=82 | 年龄为53.6（45.5，58.7）岁 | 局部晚期宫颈癌 | toripalimab **+** platinum-based concurrent chemoradiotherapy **+** consolidation chemotherapy | PD-L1 CPS < 1 n=67 (81.7%) CPS ≥1 n= 2 (2.4%) | 76（92.7%）例为鳞状细胞癌，6（7.3%）例为腺癌 | **盆腔CCRT：**放疗第1周的第1天，在静脉滴入同步化疗药前给予特瑞普利单抗注射液，240 mg，静脉输注，每21天为1个周期，共2次；含铂方案化疗于放疗第1周开始。具体方案为顺铂或奈达铂 40 mg/m²，每周1次，共4~6次 ［ 13 ］ 。肿瘤及淋巴引流区计划靶区总剂量为45.0~50.4 Gy/25~28次，淋巴结转移灶总剂量为59.92 Gy/28次。 **近距离治疗：**近距离放疗总剂量为28.0 Gy/4次，第5~8周。单纯特瑞普利单抗注射液，240 mg，静脉输注，共1次。 **放疗后巩固化疗联合免疫治疗：**在放疗结束后2周给予6周期化疗，具体剂量为：特瑞普利单抗注射液，240 mg，静脉输注（化疗前给药，第1天）及紫杉醇175 mg/m 2，第1天，顺铂75 mg/m 2，第2~4天或奈达铂80 mg/m 2，第2~3天。每3周重复1次，共6次。 |
| Camrelizumab Plus Apatinib in Patients With Advanced Cervical Cancer (CLAP): A Multicenter, Open-Label, Single-Arm, Phase II Trial | Chunyan Lan ,et al.,2020 | China | Between January 21 and August 1, 2019 | Ⅱ | Median follow-up was 11.3 months (range, 1.0-15.5 months) | n=45 | Median age was 51.0 years (range, 33-67 years) | Advanced Cervical Cancer | camrelizumab plus apatinib | PD-L1 positive n=30(66.7%) , negative n=10(22.2%) | Squamous cell carcinoma n=30(66.7%)  Adenocarcinoma n=15(33.3%) | camrelizumab 200 mg every 2 weeks and apatinib 250 mg once per day |
| A multicenter phase 2 trial of camrelizumab plus famitinib for women with recurrent or metastatic cervical squamous cell carcinoma | Lingfan Xia ,et al.,2022 | China | 2019-2020 | Ⅱ | Median follow-up lasts for 13.6 months (interquartile range: 10.0–23.6) | n=33 | Age 50（43-55） | recurrent or metastatic cervical squamous cell carcinoma | camrelizumab plus famitinib | PD-L1 CPS ≥ 1 n=33 | Squamous cell carcinoma n=33 | camrelizumab 200 mg intravenously on day 1 of each 3-week cycle plus famitinib 20 mg orally once daily. |
| NCT05247619 | Wen Q. ,et al.,2025 | China | 2022-2023 | II(single arm) | Median follow-up duration was 21.5 months | n=51 | Median (range) age of patients was 54.0 years (27–73 years) | persistent, recurrent, or metastatic cervical cancer | tislelizumab plus bevacizumab plus chemotherapy | PD-L1 CPS < 5% n=6 (11.8%) CPS = (5-20%) n= 11 (21.6%) CPS ≥ 20% n=24 (47.1%) | Squamous cell carcinoma n=47（92.2%）  Adenocarcinoma n=4 (7.8%) | tislelizumab (200 mg), bevacizumab (7.5 mg/kg), paclitaxel (175 mg/m2), and cisplatin (50 mg/m2) or carboplatin (AUC = 5) on day 1 of cycle 1 (every 3 weeks) in safety run-in stage |
| JS001-ISS-CO214 | Chen Li ,et al.,2024 | China | 2021-2022 | II(single arm) | The median follow-up duration was 18.6 (range, 3.3–28.5) months | n=24 | Age  ≥ 18 | refractory recurrent or metastatic cervical cancer | Toripalimab plus bevacizumab , chemotherapy | Not mentioned | Squamous cell carcinoma n=19 （79.2%）  Adenocarcinoma n=5 （20.8%） | toripalimab, 240 mg; bevacizumab, 7.5 mg/kg; paclitaxel, 175 mg/m2; and the investigator’s choice of cisplatin, 50 mg/m2 or carboplatin, (area under curve=5). |
| CheckMate 358 | Oaknin et al., 2024 | Spain | 2015-2020 | I-Ⅱ | NIVO3 plus IPI1 : Median follow-up times were 12·6 months (7·8–37·1) | n=45 | Age  ≥ 18 | recurrent or metastatic cervical cancer | NIVO3 plus IPI1 | PD-L1 CPS ≥ 1% n=35 (100%) CPS < 1% n= 2 (0%) CPS ≥ 10% n=25 (88%) CPS < 10% n=12 (12%) | Squamous cell carcinoma n=all | nivolumab 3 mg/kg every 2 weeks plus ipilimumab 1 mg/kg every 6 weeks , followed by nivolumab 240 mg every 2 weeks |
|  |  |  |  |  | NIVO1 plus IPI3 : Median follow-up times were 16·7 months (7·2–27·5) | n=112 |  |  | NIVO1 plus IPI3 | PD-L1 CPS ≥ 1% n=60 (100%) CPS < 1% n= 7 (0%) CPS ≥ 10% n=36 (88%) CPS < 10% n=31 (12%) |  | nivolumab 1 mg/kg every 3 weeks plus ipilimumab 3 mg/kg every 3 weeks for four cycles , followed by nivolumab 240 mg every 2 weeks |
|  |  |  |  |  | nivolumab : Median follow-up times were 19·9 months (IQR 8·2–44·8) | n=19 |  |  | nivolumab monotherapy | PD-L1 CPS ≥ 1% n=16 (100%) CPS < 1% n= 0 (0%) CPS ≥ 10% n=14 (88%) CPS < 10% n=2 (12%) |  | nivolumab 240 mg every 2 weeks |

**Supplementary Table S3. Results of included trials of ICIs in advanced CC**

| **Study Name** | **Administered Drugs** | **Nr. of patients** | **Result** | | | | | | | **Most adverse event** | **Response Assessment Method** |
| --- | --- | --- | --- | --- | --- | --- | --- | --- | --- | --- | --- |
|  |  |  | **mOS** | **ORR** | **CRR** | **mPFS** | **DCR** | **mDoR** | **Safety** |  |  |
| CALLA | Experimental arm:durvalumab + SoC CCRT | n=385 | NA , HR=0.78（95% CI 0.55 - 1.10) | 83% (318/385) OR 1·15 [95% CI 0·80-1·66] | NO | NA , HR( 0.84; 95%CI 0.65 - 1.08; p= 0.17) 24MonthsPFSR:65.9% 12MonthsPFSR:76% | ≥3 months （90.1%）  ≥6 months （84.2% ） ≥9 months （80.3%）  ≥12 months （77.5%） | No | AEs 379/385 (98%) , G3-4 AEs 199/385 (52%) Led to treatment discontinuation TRAEs 48/385 (12%) | Serious adverse events n=106 (28%) | RECIST 1.1 |
|  | Control arm: placebo + SoC CCRT | n=385 |  | 81% (310/385) OR 1·15 [95% CI 0·80-1·66] | NO | NA , HR (0.84; 95%CI 0.65 - 1.08; p= 0.17) 24MonthsPFSR:62.1% 12MonthsPFSR:73.3% | ≥3 months （ 92.4%） ≥6 months （ 85.6%） ≥9 months （80.2%） ≥12 months （76.2%） | No | AEs 377/384 (98%) , G3-4 AEs196/384（51%） Led to treatment discontinuation TRAEs 37/384 (10%) | Serious adverse events n=89(23%) |  |
| （ENGOT-cx11/GOG-3047/KEYNOTE-A18） | Experimental arm:pembrolizumab + CCRT | n=529 | NA , 24 OS=87% (82–91) DHR 0·73 (0·49–1·07) | 79% (413 /521) | NO | PFS Event:115/529 , 24MonthsPFSR:68% Median (95% CI), months ：NR (25·0-NR) | NO | NA ,12 months or longer 172 (81%) | AEs : 525/528 (99%) , ≥G3 AEs 394/528 (75%)Led to treatment discontinuation TRAEs 81/528 (15%) | Serious adverse events n= 91/528 (17%) | RECIST 1.1 |
|  | Control arm: placebo + CCRT | n=531 | NA , 24OS=81% (75–86) DHR 0·73 (0·49–1·07) | 76%（396/522） | NO | PFS Event:154/531 , 24MonthsPFSR:57% Median (95% CI), months ：NR (24·8-NR) |  | NA ,12 months or longer 153 (77%) | AEs: 526/530 (99%)，≥G3 AEs 364/530 (69%) Led to treatment discontinuation TRAEs 67/530 (13%) | Serious adverse events n= 65/530 (12%) |  |
| BEATcc/ENGOT-cx10 | Experimental arm: Atezolizumab + chemotherapy+Bevacizumab | n=206 | Experimental arm: 32.1 mos(105/206); Control arm: 22.8 mos(129/204) Events/patients: Ad:55/89; Sq: 179/321 | 84% (CR: 32%) | NO | Experimental arm: 13.7mos (138/206); Control arm: 10.4 mos(166/204) Events/patients: Ad:73/89; Sq: 231/321 | NA | 13.6 mos | TRAEs 88.3%, ≥G3 AEs 79.0% | Asthenia, Nausea, Alopecia, Neutropenia, Anemia, Constipation, Diarrhea, Hypertension | RECIST 1.1 |
|  | Control arm: Chemotherapy+Bevacizumab(standard therapy) | n=204 |  | 72% (CR: 20%) | NO |  | NA | 8.6 mos | TRAEs 88.3%, ≥G3 AEs 75.0% |  |  |
| KEYNOTE-826 | Experimental arm:pembrolizumab+platinum-based chemotherapy+bevacizumab | n=308 | PD-L1 CPS≥1: NA (24mOS=53%) Intention-to-treat : 24.4mOS (24mOS=50.4%) PD-L1 CPS≥10 : NA (24mOS=54.4%) | PD-L1 CPS≥1: 185/273 (68.1%) Intention-to-treat :202/308 (65.9%) PD-L1 CPS≥10 :109/158 (69.6%) | PD-L1 CPS≥1: 22.7% Intention-to-treat : 21.4% PD-L1 CPS≥10 : 22.2% | PD-L1 CPS≥1: 10.4mos [95% confidence interval {CI}, 9.7 to 12.3] Intention-to-treat : 10.4mos [95%CI，9.1-12.1] PD-L1 CPS≥10 : 10.4mos [95%CI，8.9-15.1] | NO | PD-L1 CPS≥1: 18.0 mos Intention-to-treat : 18.0 mos PD-L1 CPS≥10 : 21.1 mos | ≥G3 AEs 251/307 （81.8%） | Serious adverse events 152/307 (49.8%) | RECIST 1.1 |
|  | Control arm:placebo+platinum-based chemotherapy+bevacizumab | n=309 | PD-L1 CPS≥1: NA (24mOS=41.7%) Intention-to-treat :16.3-16.5mOS (24mOS=40.4%) PD-L1 CPS≥10 : NA (24mOS=44.6%) | PD-L1 CPS≥1: 155/275 (50.2%) Intention-to-treat : 156/309 (50.8%) PD-L1 CPS≥10 : 78/159 (49.1%) | PD-L1 CPS≥1: 13.1% Intention-to-treat : 12.9% PD-L1 CPS≥10 : 11.3% | PD-L1 CPS≥1: 8.2mos [95%CI，6.3-8.5] Intention-to-treat : 8.2mos [95%CI，6.4-8.4] PD-L1 CPS≥10 : 8.1mos [95%CI，6.2-8.8] |  | PD-L1 CPS≥1: 10.4 mos Intention-to-treat : 10.4 mos PD-L1 CPS≥10 : 9.4 mos | ≥G3AEs 232/309 (75.1%) | Serious adverse events 131/309 (42.4%) |  |
| COMPASSION-16 | Experimental arm: Cadonilimab + platinum-based chemotherapy + bevacizumab | n=222 | NA [95% CI 27·0 to not estimable] ，24mOS: 62·2% (55·2–68·5) PD-L1 CPS≥1: NA (95% CI 26·6 to not estimable) PD-L1 CPS<1: NA (23·0 to not estimable) Bevacizumab user :NA (27·8 to not estimable ) Without bevacizumab :28·2 mos (17·5 to not estimable) | 184/222 (83%) | NO | 12.7mos PD-L1 CPS≥1: 13.5mos（95% CI 10.8 - 17.3） PD-L1 CPS<1 : 12·0 mos (9·5–14·5) Bevacizumab user :15·1 mos (11·8–19·7) Without bevacizumab :11·7 mos (9·5–14·5) | (208/222) (94%) | 13·2 mos (IQR 6·2 to not estimable) | AEs 225/226(>99%), ≥G3AEs 193/226 TRAEs 225/226(99%), ≥G3TRAEs 186/226(82%) | NO | RECIST 1.1 |
|  | Control arm: platinum-based chemotherapy + bevacizumab | n=223 | 22·8 mos [17·6–29·0] , 24mOS: 48·4% (41·1–55·4) PD-L1 CPS≥1: 22·7 mos (15·5 to not estimable) PD-L1 CPS<1: 25·3 mos (17·7 to not estimable) Bevacizumab user ：NA (25·7 to not estimable) Without bevacizumab :15·1 mos (12·2 to 17·6) | 153/223 (69%) | NO | 8.1mos PD-L1 CPS≥1: 8·3 months (7·5–9·7) PD-L1 CPS<1 :8·2 months (7·8–11·8) Bevacizumab user ：11·5 months (9·5–14·8） Without bevacizumab :6·9 mos (5·6–7·8) | (205/223) (92%) | 8·2 months (4·7 to 25·7) | AEs 219/219(100%), ≥G3AEs 176/219 TRAEs 219/219(100%), ≥G3TRAEs 173/219(79%) | NO |  |
| SKYSCRAPER-04 | Experimental arm: Tiragolumab + Atezolizumab | n=126 | 11.1mos (95%CI 9.6-14.5) | 19.0% (95%CI 12.6-27.0) | 3.2%（95%CI 0.9-7.9） | 2.8mos (95%CI 1.7 - 4.1) | 31.0%（95%CI 23.0-39.8） | 11.8 months (95% CI 6.7 to not estimable) | TRAEs 83/126 (66%) , ≥G3TRAEs 17/126 (13%) AEs 118/126 (94%) , ≥G3AEs 55/126 (44%) | Serious adverse events 37/126 (29%) | RECIST 1.1 |
|  | Control arm: Atezolizumab | n=45 | 10.6mos (95%CI 6.9-13.8) | 15.6% (95%CI 6.5-29.5) | 4.4%（95%CI 0.5-15.1） | 1.9mos (95%CI 1.5 - 3.0) | 20.0%（95%CI 9.6-34.6） | not estimable | TRAEs 23/45 (55%) , ≥G3TRAEs 14/45(31%) AEs 41/45 (91%) , ≥G3AEs 4/45 (9%) | Serious adverse events 12/45 (27%) |  |
| IBI310 plus sintilimab vs. placebo plus sintilimab in recurrent/metastatic cervical cancer: A doubleblind, randomized controlled tria | Experimental arm: IBI310 plus sintilimab | n = 103 | 13.9 mos (95% CI: 11.5–25.6) | 32.3% (95% CI：23.3%-42.5%) | NO | 3.6 mos ( 95% CI: 2.7–6.3) | 30/99 (30.3%) | NA | TRAEs : 97/103 (95%) , ≥G3TRAEs 56/103 (55%) | NO | RECIST 1.1 |
|  | Control arm: placebo plus sintilimab | n = 102 | 17.2 mos (95% CI: 13.7– 25.9) | 23.5% (95% CI：15.5%-33.1%) |  | 4.2 mos (95% CI: 2.8–6.2) | 34/98 (34.7%) | 12.6 months (95% CI: 10.0– NR) | TRAEs : 75/102 (76%) , ≥G3TRAEs 19/102 (19%) |  |  |
| AdvanTIG-202 | Experimental arm: TIS + OCI | n=138 | Cohort 1 : 12.2 mos(9.9–16.6) PD-L1+ : 16.4 mos(10.4–NE) PD-L1- : 10.3 mos (8.1–13.4) | Cohort 1 : 23.2% (16.4–31.1) PD-L1+ : 27.4% (18.2–38.2) PD-L1- : 17.0% (8.1–29.8) | Cohort 1 : 15/138 (10.9%) PD-L1+ : 12/84 (14.3%) PD-L1- : 3/53 (5.7%) | Cohort 1 : 3.0 mos (2.6–4.9) PD-L1+ : 4.1mos (2.7–6.9) PD-L1- : 2.6 mos (1.5–4.3) | NO | Cohort 1 : 17.3 mos (16.9–NE) PD-L1+ : 16.9 mos (16.9–NE) PD-L1- : NE mos (2.8–NE) | TRAEs : 97/138 (70.3%) , ≥G3TRAEs :25/138 (18.1%) | NO | RECIST 1.1 |
|  | Control arm: TIS | n=40 | 23.5 months (95% CI 13.6 months–NE) | 14 /40 (35.0% [95% CI 20.6%–51.7%]) (5 CR/9 PR) PD-L1+ : 7/20 (35.0% [95% CI 15.4%–59.2%]) (5 CR/2 PR) PD-L1- : 7/20 (35.0% [95% CI 15.4%–59.2%]) (0 CR/7 PR) | NO | 5.7 mos (95% CI 2.3–8.1) | NO |  | TRAEs : 25/40 (62.5%) , ≥G3TRAEs : 2/40 (5%) |  |  |
| CheckMate 358 | NIVO3 plus IPI1 | n=45 | All treated: 15·2 mos(9·0–36·2) First-line :36·2 mos(17·1–NR) Second-line or later-line:10·3 mos(7·8–15·2) | All treated: 14/45 (31%; 18–47) First-line : 7/18 (39%; 17–64) Second-line or later-line: 7/27 (26%; 11–46) | NO | All treated: 3·8 mos(2·1–10·3) First-line : 17·1 mos(2·1–36·4) Second-line or later-line:3·6 mos(1·8–5·1) | NO | All treated : 24·4 mos(8·7–NR) First-line : 34·6 mos(6·6–NR) Second-line or later-line :21·1mos(7·5–NR) | TRAEs : 36/45 (80%) , ≥G3TRAEs :13/45 (29%) | Serious treatment-related adverse events: 47/112(42%) | RECIST 1.1 |
|  | NIVO1 plus IPI3 | n=45 | All treated: 24·7 mos(16·6–49·1) First-line : 27·4 mos(13·9–NR) Second-line or later-line:24·7 mos(13·0–NR) | All treated: 18/45 (40%; 26–56) First-line : 12/25 (48%; 28–69) Second-line or later-line: 6/20 (30%; 12–54) | NO | All treated : 7·2 mos(3·8–17·2) First-line :8·8 mos(3·7–35·9) Second-line or later-line : 5·8 mos(2·0–14·1) | NO | All treated :34·1mos (15·3–NR) First-line : 34·1mos (5·8–NR) Second-line or later-line :NR (2·6–NR) | TRAEs :99/112 (88%) , ≥G3TRAEs : 52/112 (46%） | Serious treatment-related adverse events: 12/45 (27%) | RECIST 1.1 |
|  | NIVO1 plus IPI3 | n=112 | All treated:20·9 mos(14·4–32·8) First-line : 20·9 mos(13·9–NR) Second-line or later-line:19·9 mos(7·8–32·8) | All treated: 43/112 (38%; 29–48) First-line : 28/69 (41%; 29–53) Second-line or later-line: 15/43 (35%; 21–51) | NO | All treated : 5·8 mos(3·8–9·3) First-line : 7·0 mos(3·8–10·4) Second-line or later-line : 4·7 mos(3·2–10·0) | NO | All treated : 34·1mos(11·5–NR) First-line : 25·6 mos(9·2–NR) Second-line or later-line : NR mos(5·2–NR) |  |  |  |
|  | nivolumab monotherapy | n=19 | All treated: 21·6 mos (8·3–46·9) First-line : NA Second-line or later-line:21·9 mos(8·3–NR) | All treated: 5/19 (26%; 9–51) First-line : 1/4 (25%; 1–81) Second-line or later-line: 4/15 (27%; 8–55) | NO | All treated: 5·1mos (1·9–9·1) First-line : NA Second-line or later-line: 5·5mos (1·8–9·1) | NO | All treated : NR (35·3–NR) First-line : NA Second-line or later-line : NR | TRAEs : 12/19 (63%) , ≥G3TRAEs : 4/19 (21%） | Serious treatment-related adverse events: 3/19 (16%) | RECIST 1.1 |
| QL1604 plus paclitaxel-cisplatin/carboplatin in patients with recurrent or metastatic cervical cancer: an open-label, single-arm, phase II trial | QL1604 + paclitaxel-cisplatin/carboplatin | n=46 | NA | 58.7% (95%CI=45.4%–74.9%) PD-L1 CPS≥10 :73.3% (11/15) PD-L1 CPS<10 : 51.6% (16/31) | NO | 8.5 mos (95%CI 6.8–15.1 mos) | 89.1% (95% CI=76.4%–96.4%) | 9.6 mos PD-L1 CPS≥1: 11.2 mos | TRAEs : 45/46 (97.8%) , ≥G3TRAEs : 36/46 (78.3%） | NO | RECIST 1.1 |
| COMPASSION-13 | cohort A-10 :cadonilimab 15 mg/kg every 3 weeks+chemotherapy | n=15 | NA 12-mos 87.5% (95% CI, 58.6–96.7) | 68.8%（11/16；95%CI, 41.3 - 89.0） | NO | 7.06 mos (95% CI, 4.1–NE) 12-mos 37.5% (95% CI, 15.4–59.8) | 100% | 7.62 mos (95% CI, 2.86–NE) | TRAEs :16/16 (100.0%) , ≥G3TRAEs :12/16 (75.0%) Cadonilimab-related grade ≥ 3 TRAEs : 11/16 (68.8%) | NO | RECIST 1.1 |
|  | cohort A-15 :cadonilimab 10 mg/kg every 3 weeks+chemotherapy | n=12 | NA 12-mos: 93.3% (95% CI, 61.3–99.0) | 66.7%（10/15;95% CI，38.4–88.2） | NO | 11.10 mos (95% CI, 5.5–NE) 12-mos 49.0% (95% CI, 21.6–71.7) |  | NA | TRAEs : 15/15(100.0%) , ≥G3TRAEs : 9/15 (60.0%) Cadonilimab-related grade ≥ 3 TRAEs : 7/15 (46.7%) | NO | RECIST 1.1 |
|  | cohort B-10 :cadonilimab 10 mg/kg every 3 weeks+chemotherapy+bevacizumab | n=13 | NA | 92.3%（12/13; 95% CI, 64.0 - 99.8） | NO | NA |  | NA | TRAEs :14/14 (100.0%) , ≥G3TRAEs : 12/14 (85.7%) Cadonilimab-related grade ≥ 3 TRAEs :7/14 (50.0%) | NO | RECIST 1.1 |
| NICOL | Nivolumab plus chemoradiotherapy | n=16 | NO | 93.8% (95% (CI)：69.8–99.8%) Last follow-up:75% ( 95% (CI) : 47.6;92.7) | 8/16 (50%) | NA 2years :75% (95%CI : 56.5–99.5%) | NO | NA | G4TRAEs:5/16 (31.2%) Following the 11-week DLT assessment ≥G3 AE 3/16 (12.5%) | NO | RECIST 1.1 |
| TRACE | CCRT plus toripalimab | n=22 | 24 mOS : 90.9% | 100% | 20/22 (90.9%) PD-L1 CPS ≥ 5 CR : 100% (7/7) PD-L1 CPS < 5 :86.7% (13/15) | 24 mos: 81.8% | NO | NA | AEs: 22/22 (100%) , ≥G3AEs : 11/22 (50%) | NO | RECIST 1.1 |
| A prospective phase Ⅱ clinical trial of toripalimab combined with platinum-based concurrent chemoradiotherapy and consolidation chemotherapy in patients with locally advanced cervical cancer | toripalimab + platinum-based concurrent chemoradiotherapy + consolidation chemotherapy | n=82 | 12 OS :98.6% , 24 OS :95.2% , Ⅲ ~Ⅳ12 OS : 98.4% , 24 OS :96.3% PD-L1 CPS≥10 : 97.3% ,CPS<10 : 94.4% | 72/82 (87.8%) Ⅲ ~Ⅳ 63/69 (91.3%) | 64/82（78.0%） | NA 24Mos:88.4% 12Mos:90.8% Ⅲ ~Ⅳ 24Mos:90.8% , 12Mos:94.0% 24mos PD-L1 CPS≥10 : 92.4% ,CPS<10 : 81.2% | 72/82 (87.8%) Ⅲ ~Ⅳ 63/69 (91.3%) | NA | TRAEs :37/82 (45.1%) , ≥G3TRAEs :17/82 (20.7%) | NO | RECIST 1.1 |
| Camrelizumab Plus Apatinib in Patients With Advanced Cervical Cancer (CLAP): A Multicenter, Open-Label, Single-Arm, Phase II Trial | camrelizumab plus apatinib | n=45 | NA (95% CI, 11.6 months to not estimable) 9-mos 69.2% (95% CI, 52.9% to 80.8%) | 25/45（55.6%[95%CI，40.0%-70.4%]） | NO | 8.8 mos (95% CI, 5.6 months to not estimable) 6 mos: 57.0% (95% CI, 40.2% to 70.7%) | 82.2%（95%CI，67.9%- 92.0%) | Confirmed objective response 25 : NA (95% CI, 5.6 mos to not estimable） 6mos: 71.5% (95% CI, 49.3% to 85.3%) 12mos: 66.8% (95% CI, 44.2% to 81.9%) | TRAEs : 43/45(95.6%) , ≥G3TRAEs :32/45 (71.1%) | NO | RECIST 1.1 |
| A multicenter phase 2 trial of camrelizumab plus famitinib for women with recurrent or metastatic cervical squamous cell carcinoma | camrelizumab plus famitinib | n=33 | NA , 6mos: 93.9% (95% CI: 77.9–98.4) 9mos: 84.2% (95% CI: 66.1–93.1) 12mos: 77.7% (95% CI: 58.9–88.7) | 13/33 39.4% ( 95% [CI]: 22.9–57.9) | NO | 10.3 mos (95% CI: 3.5–not reached) 6mos: 59.5% (95% CI: 39.8–74.7) 9mos:52.5% (95% CI: 33.3–68.6) 12mos: 49.0% (95% CI: 30.1–65.4) | 23/33 69.7% ( 95% CI: 51.3–84.4) | NA (95% CI: 8.2–not reached) 6mos: 100.0%（95%CI: 100.0–100.0） 9mos: 83.3% (95% CI: 48.2–95.6) 12mos: 74.1% (95% CI: 39.1–90.9) | TRAEs :33/33 (100%) , ≥G3TRAEs :26/33 (78.8%) | Serious treatment-related adverse events: 9/33 (27.3%) | RECIST 1.1 |
| NCT05247619 | tislelizumab plus bevacizumab plus chemotherapy | n=51 | NA 2-year OS 75.6% (95% CI: 58.4, 86.5) | Total:83.0% (95% CI 69.2-92.4) EAS :91.5% (95% CI: 79.6- 97.6) | NO | 22.6 mos (95% CI: 14.6, not reached) 12-mos 77.9% (95% CI: 61.8, 87.8) | NO | NA 18-mos DOR: 51.5% (95% CI: 31.0, 68.7) | TRAEs :50/51 (98.0%) , ≥G3TRAEs :28/51 (54.9%) | Serious treatment-related adverse events: 12/51 (23.5%) | RECIST 1.1 |
| JS001-ISS-CO214 | Toripalimab plus bevacizumab , chemotherapy | n=24 | NA (95% CI=not reached) | 83.3% (95% CI=62.6–95.3) | NO | 22.6 mos (95% CI=10.4–34.7) | 95.8% (95% CI, 78.9–99.9) | NA | TRAEs :22/24 (91.7%) , ≥G3TRAEs :14/24 (58%) | NO | RECIST 1.1 |

**Supplementary Table S4. Risk of bias for the 11 included publications (single arm studies), based on the Newcastle-Ottawa Scale, NOS**

| Author**，**year | **Type of bias** | | | | | | | | **Total** |
| --- | --- | --- | --- | --- | --- | --- | --- | --- | --- |
|  | **Is the case definition adequate?** | **Representativeness of the cases** | **Selection of Controls** | **Definition of Controls** | **Comparability of cases and controls on the basis of the design or analysis** | **Ascertainment of exposure** | **Same method of ascertainment for cases and controls** | **Non-Response rate** |  |
|  |  |  |  |  |  |  |  |  |  |
|  |  |  |  |  |  |  |  |  |  |
|  |  |  |  |  |  |  |  |  |  |
|  |  |  |  |  |  |  |  |  |  |
|  |  |  |  |  |  |  |  |  |  |
|  |  |  |  |  |  |  |  |  |  |
| AdvanTIG-202 2025 | ***** | * | * | * | * | * | * | * | 8 |
| CheckMate 358 2024 | ***** | * | * | * | ** | * | * | * | 9 |
| SKYSCRAPER-04 2023 | ***** | * | * | * | ** | * | * | * | 9 |
| COMPASSION-13 2024 | ***** | * | * | * | ** | * | * | * | 9 |

**Supplementary Table S5. Risk of bias for the 11 included publications (single arm studies), based on the ROBINS-I tool (low, moderate, serious, critical)**

| **Author，year** | Bias due to confounding | Bias due to selection of participants | Bias due to exposure  assessment | Bias due to misclassification during follow-up | Bias due to measurement of the outcome | Bias due to missing data | Bias due to selective reporting of the results | Overall rating |
| --- | --- | --- | --- | --- | --- | --- | --- | --- |
|  |  |  |  |  |  |  |  |  |
|  |  |  |  |  |  |  |  |  |
|  |  |  |  |  |  |  |  |  |
| C. Fang 2024 | moderate | moderate | low | low | moderate | moderate | low | moderate |
| NICOL 2023 | moderate | moderate | low | low | low | low | low | low |
| D. Ou 2024 | moderate | moderate | low | low | low | low | low | moderate |
| J. Chen 2024 | moderate | moderate | low | low | low | low | low | low |
| C. Lan 2020 | moderate | low | low | low | low | low | low | low |
| L. Xia 2022 | moderate | moderate | low | low | low | low | low | low |
| Q. Wen 2025 | moderate | moderate | low | low | low | low | low | moderate |
| C. Li 2025 | moderate | moderate | low | low | low | low | low | moderate |
